# HIV infection and multidrug resistant tuberculosis: a systematic review and meta-analysis

**DOI:** 10.1186/s12879-020-05749-2

**Published:** 2021-01-11

**Authors:** Zeeba Zahra Sultana, Farhana Ul Hoque, Joseph Beyene, Md. Akhlak-Ul-Islam, Md Hasinur Rahman Khan, Shakil Ahmed, Delwer Hossain Hawlader, Ahmed Hossain

**Affiliations:** 1 CAPABLE- A Cambridge-led program in Bangladesh, University of Cambridge, Cambridge, UK; 2 Department of Public Health, North South University, Dhaka, Bangladesh; 3 Department of Health Research Methods, Evidence, and Impact, Faculty of Health Sciences, McMaster University, Hamilton, Ontario Canada; 4 Department of Hematology, Bangabandhu Sheikh Mujib Medical University, Dhaka, Bangladesh; 5 Institute of Statistical Research and Training, University of Dhaka, Dhaka, Bangladesh; 6 Global Health Institute, North South University, Dhaka, Bangladesh; 7 Health Management BD Foundation, Dhaka, Bangladesh

**Keywords:** Multidrug resistant, Drug-resistant, Tuberculosis, MDR-TB, HIV, Meta-analysis

## Abstract

**Background:**

Multidrug-resistant tuberculosis (MDR-TB) in HIV infected individuals is a serious threat to global efforts to combat tuberculosis. Inconsistent findings on the association between HIV infection and MDR-TB were present in many studies. We aimed to review existing data on the relationship between HIV infection and MDR-TB systematically to assess the contribution of HIV on MDR-TB worldwide. We also investigated the patterns of MDR-TB by age, country-wise income, study designs, and global regions.

**Methods:**

We utilized PubMed, Google Scholar, and ScienceDirect databases to select eligible studies for meta-analysis that were published between January 1, 2010, and July 30, 2020. The random-effects model was used to obtain the pooled odds ratio of the crude association between HIV and MDR-TB with a 95% confidence interval. We investigated the potential publication-bias by checking funnel plot asymmetry and using the Egger’s test. Moreover, we assessed the heterogeneity using the *I*^*2*^ statistic. Sensitivity analysis was performed based on sample size and adjustment factors. The protocol was registered with PROSPERO-CRD42019132752.

**Results:**

We identified 1603 studies through a database search, and after subsequent eliminations we selected 54 studies including 430,534 TB patients. The pooled odds of MDR-TB was 1.42 times higher in HIV-positive patients than HIV-negative patients (OR=1.42,CI=1.17–1.71, *I*^*2*^=75.8%). Subgroup analysis revealed that the estimated pooled odds for South-East Asian countries was 1.86, which is the highest in WHO regions (OR=1.86,CI=1.30–2.67, *I*^*2*^=0.00%), followed by Europe and Africa. The effect estimate was found to be higher for primary MDR-TB (OR=2.76,CI=1.70–4.46, *I*^*2*^=0.00%). There was also a trend towards increased odds of MDR-TB for HIV patients older than 40 years (OR=1.56,CI=1.17–2.06). The association was found to be significant in high-burden TB countries (OR=1.75, CI=1.39–2.19) and in high-income countries (OR=1.55, CI=1.06–2.27).

**Conclusion:**

Such findings indicate that HIV infection raises the risk of MDR-TB, and after contrasting it with the results of the earlier pooled study, it appeared to be an upward risk trend. Moreover, we found that the risk is the highest in the South-East Asian region. A balanced allocation of resources is needed to halt both primary and secondary MDR-TB, particularly in HIV infected people with 40 years of age and older.

**Supplementary Information:**

The online version contains supplementary material available at 10.1186/s12879-020-05749-2.

## Background

Tuberculosis is a global public health problem causing illness among millions each year. In 2018, there were an estimated 10 million new TB cases, and 8.6% were living with HIV [1]. The care and control of tuberculosis are threatened by the emergence and amplification of multi-drug resistant tuberculosis (MDR-TB). It was declared as a public health crisis by WHO in 2013 [2]. Globally, there were an estimated 484,000 incident cases of MDR/Rifampicin-resistant tuberculosis (RR-TB). Of them, about 39% (186772) were notified, and 32% (156071) enrolled for treatment in 2018 [1]. The large gap in diagnosis and treatment increases the likelihood of transmission of TB plus MDR-TB [3]. Poor TB treatment outcome is also reported in TB-HIV co-infected patients, which further ignites MDR-TB development [4–6]. A WHO report shows that 3.4% of new cases and 18% of previously treated TB cases are estimated to have MDR/RR-TB [1]. However, HIV positive individuals are 20 times more likely to develop active TB than those without HIV [7]. Despite enormous concerted measures taken to control tuberculosis and reduce HIV-associated deaths, it ranks among the top ten causes of death worldwide [1]. About 0.25 million deaths were attributed to TB associated with HIV, and nearly 15% of all global tuberculosis deaths were contributed by MDR-TB in 2018 [1].

Drug-resistant tuberculosis (DR-TB) is defined as a case of one or more anti-TB drugs resistant to bacteria that cause TB. There are various forms of DR-TB: mono-drug-resistant TB (mono-DR-TB), polydrug-resistant TB (poly-DR-TB), rifampicin-resistant TB (RR-TB), multidrug-resistant TB (MDR-TB), and extensively drug-resistant TB (XDR-TB). MDR-TB is a form of TB that does not respond to at least isoniazid and rifampicin, the two most potent anti-TB drugs. The XDR-TB is also a form of MDR-TB resistant to isoniazid and rifampicin, plus any fluoroquinolone and at least one of three injectable second-line drugs (i.e., amikacin, kanamycin, or capreomycin). RR-TB is classified as a case of TB, which shows either rifampicin (R) resistance only or accompanied by resistance to other anti-TB drugs in different forms. About half a million people worldwide acquired MDR-TB in 2017, and a further 161,000 people with RR-resistant TB were newly diagnosed with MDR-TB [1]. China, India, and Russia were the countries with the largest number of MDR / RR-TB cases (47% of the global total). Approximately 6.2% of these cases were XDR-TB [1].

Global tuberculosis burden study 2015 suggests taking the necessary steps to prevent HIV for reducing the burden of tuberculosis [8]. Estimates reveal that around 1.7 billion of the world’s population has latent tuberculosis infection [1]. Of them, 5–15% of cases are at risk of developing active diseases during their lifetime, and people living with HIV have a higher risk of falling ill [9, 10]. Alarmingly, it appeared from a mathematical model that there were approximately 19 million people globally with latent MDR tuberculosis infection (10% were at risk of active disease) in 2015 [11]. Undoubtedly, latency is affected by HIV infection and is one of the most potent risk factors for converting into active disease, drug-susceptible, or drug-resistance [10–12].

The surge of MDR-TB occurrence in HIV-prevalent settings is of great public health importance. An updated understanding of the magnitude of association is needed with the accumulation of recent evidence that supports a positive association between HIV and MDR-TB [13, 14]. Additionally, two systematic reviews revealed an increased risk of transmission-associated MDR-TB (primary) among HIV positive individuals [15, 16]. Mesfin et al. conducted the latest systematic review and meta-analysis on the relationship between HIV infection and MDR-TB in 2014, which included research from 1994 to 2011. Even after the latest meta-analysis, conflicting results on the association were found in numerous studies conducted during the last decade. The objective was to provide an up-to-date pooled risk estimate on the relationship between HIV infection and MDR-TB growth and compare the risk pattern of MDR-TB with previous systematic reviews.

## Methods

### Search strategy

We researched on PubMed, Google Scholar, and Science Direct databases to select eligible peer-reviewed articles for our systematic review and meta-analysis. Relevant observational studies (cross-sectional, case-control, and cohort) were screened to assess the association between HIV infection and MDR-TB, published from January 1, 2010, to July 30, 2020 (date of the last search). The screening language was restricted to English. The particular keywords used for searching articles were “Multidrug-resistant tuberculosis” or “MDR-TB” or “Drug-resistant TB” or “Risk factors of MDR-TB” or “Predictors of MDR-TB” and “HIV” or “Human Immunodeficiency Virus”. A summary of search terms is provided in Appendix A. Search results were compiled using a citation management software Zotero. In addition to databases used, we explored references of selected studies to incorporate all potential pertinent articles to construct our summary estimates. The study adheres to the Preferred Reporting Items for Systematic Review and meta-analysis (PRISMA) guidelines [17]. The protocol was registered in the PROSPERO database-CRD42019132752.

### Selection criteria

The primary outcome of the meta-analysis was an unadjusted odds ratio exhibiting the crude association between HIV infection and MDR-TB. The articles that reported or provided adequate data to calculate the frequency of MDR-TB and non-MDR-TB, subdivided by HIV status (positive or negative), were included in our systematic review. We included studies where MDR-TB is the outcome of interest. We contacted the author for the insufficient information given in any study. If the author didn’t reply, we excluded the article. To be eligible for inclusion, any drug susceptibility testing method (culture on solid and/or liquid media, molecular techniques, clinical records, Table 1) were accepted for the diagnosis of multidrug-resistant tuberculosis (*Mycobacterium tuberculosis* strain resistant at least to rifampicin and isoniazid). The comparison group (non-MDR-TB) consisted of drug-susceptible tuberculosis patients and/or resistant to any single drug. HIV status was ascertained by HIV test reports, clinical/hospital records, databases/registers, or directly from patients. Studies reported on non-tuberculous mycobacterium, case reports, case studies, systematic review, meta-analysis, a duplicate publication of the same study, and studies with only abstract were excluded. We also didn’t include grey literature (theses and dissertations). Studies with less than three HIV/MDR-TB participants were excluded because these studies cause the analysis to be heterogeneous. Titles and abstracts of the studies obtained from database searches were screened independently by two reviewers ZZS and FH. Full text of potential articles was further reviewed for eligibility regardless of the study base (hospital or population). Articles might have been eliminated for more than one rationale. Any disagreements were discussed with senior authors (JB or AH) until a consensus was reached or by the arbitration of AH alone.

**Table 1:** Characteristics of the included studies in the meta-analysis (according to the order of year)

Author [Ref]	Year	Study design	Study period	Country	Number of TB patients	MDR-TB cases	% of male (MDR-TB cases)	Mean/ Median age (MDR-TB cases), years	TB form	% of EPTB (MDR-TB cases)	MDR-TB type	% of PTC (MDR-TB cases)
Brito et al. [18]	2010	Cross-sectional	2004–2006	Brazil	595	44	72.73	40.1	PTB, EPTB	NA	1^o^,2^o^	61.36
Andrew et al. [19]	2010	Case-control	2005–2007	South Africa	378	123	50	~ 34	PTB, EPTB	28.63	1^o^,2^o^	71.75
Sangare et al. [20]	2010	Cross-sectional	2005–2006	Burkina Faso	550	47	NA	NA	PTB	NA	1^o^,2^o^	81.03
Sangare et al. [21]	2011	Cross-sectional	2005–2006	Burkina Faso	316	42	87.66	NA	NA	NA	1^o^,2^o^	85.71
Gudo et al. [22]	2011	Cross-sectional	2007–2008	Mozambique	1102	27	61.8	~ 35	PTB	NA	1^o^	0
Vadwai et al. [23]	2011	Cross-sectional	2009	India	250	184	61.95	33.06	PTB	NA	1^o^,2^o^	62.5
Macedo et al. [24]	2012	Cross-sectional	2008–2010	Portugal	2093	50	78	~ 42	PTB, EPTB	8	1^o^,2^o^	30
Padilla et al. [25]	2012	Cross-sectional	2009–2010	Swaziland	840	122	37.70	~ 33	PTB	NA	1^o^,2^o^	77.87
van Halsema et al. [26]	2012	Cross-sectional	2002–2008	South Africa	2431	168	NA	NA	NA	NA	1^o^,2^o^	82.74
Ricks et al. [27]	2012	Case-control	2007–2009	Namibia	368	117	56	36	PTB, EPTB	2	1^o^,2^o^	97
Tessema et al. [28]	2012	Cross-sectional	2009	Ethiopia	260	13	53.85	NA	PTB	NA	1^o^,2^o^	61.54
Coelho et al. [29]	2012	Cross-sectional	2000–2004	Brazil	671	32	NA	39.2	PTB	NA	1^o^,2^o^	68.7
Ulmasova et al. [30]	2013	Cross-sectional	2010–2011	Uzbekistan	1037	372	57.07	~ 45	PTB	NA	1^o^,2^o^	NA
Minion et al. [31]	2013	Surveillance	1997–2008	Canada	15,993	177	59.35	30	PTB	NA	1^o^,2^o^	31.10
Sethi et al. [32]	2013	Cross-sectional	2006–2010	India	219	39	66.67	36.6	PTB	NA	1^o^,2^o^	69.23
Van Den Hof et al. [33]	2013	Cross-sectional	2007–2011	Kazakhstan	146,461	18,338	65.81	NA	PTB, EPTB	1.41	1^o^,2^o^	85.9
Lukoye et al. [34]	2013	Cross-sectional	2009–2011	Uganda	1537	31	67.74	~ 35	PTB	NA	1^o^,2^o^	45.16
Hang et al. [35]	2013	Cross-sectional	2007–2009	Vietnam	546	22	NA	38.6	PTB	NA	1^o^	0
Skrahina et al. [36]	2013	Cross-sectional	2010–2011	Belarus	1420	612	80	~ 46	PTB	NA	1^o^,2^o^	75.60
Satti et al. [37]	2013	Cohort	2007–2011	Lesotho	148	47	NA	NA	PTB	NA	2^o^	100
Hirpa et al. [38]	2013	Case-control	2011–2012	Ethiopia	268	134	60.50	25.1	PTB, EPTB	3	2^o^	100
Mor et al. [39]	2014	Cross-sectional	1999–2010	Israel	3552	207	75.40	43	PTB, EPTB	3.90	2^o^	100
Post et al. [40]	2014	Cross-sectional	2004–2006	Belarus, Latvia, Russia, Romania, Ukraine	144	55	74.6	30.2	PTB. EPTB	3.6	1^o^,2^o^	16.4
Metcalfe et al. [41]	2014	Cross-sectional	2011–2012	Zimbabwe	129	25	52	~ 34	PTB	NA	1^o^,2^o^	12
Shariff et al. [42]	2015	Case-control	2013–2014	Malaysia	150	30	66.70	51	PTB	NA	1^o^,2^o^	66.70
Jitmuang et al. [43]	2015	Case-control	2010–2012	Thailand	188	47	57.40	48.9	PTB, EPTB	10.60	1^o^,2^o^	53.20
Chuchottaworn et al. [44]	2015	Case-control	2007–2013	Thailand	290	145	65.50	46.2	PTB	NA	1^o^,2^o^	96.60
Elmi et al. [45]	2015	Case-control	2010–2014	Malaysia	314	105	66.70	51	PTB	NA	1^o^,2^o^	50.50
Mulisa et al. [46]	2015	Case-control	2013–2014	Ethiopia	265	88	56.80	~ 33	PTB	NA	1^o^,2^o^	90.90
Mulu et al. [47]	2015	Case-control	2013–2014	Ethiopia	306	153	57.50	35	PTB, EPTB	7.80	1^o^,2^o^	96.10
Gunther et al. [48]	2015	Case-control	2010–2011	23 TBNET sites in 16 countries in Europe	756	380	62.89	~ 36	PTB	NA	1^o^,2^o^	35
Ershova et al. [49]	2015	Cross-sectional	2012	Russia	229	44	77.30	~ 36	PTB	NA	1^o^	0
Abdella et al. [50]	2015	Cross-sectional	2012–2013	Ethiopia	70	22	NA	~ 32	PTB	0	2^o^	100
Tadasse [51]	2015	Cross-sectional	2008–2011	Ethiopia	439	113	64.60	29	PTB, EPTB	11.50	1^o^,2^o^	99.11
Salindri et al. [52]	2016	Cohort	2011–2014	Georgia	318	52	71.15	~ 50	PTB	NA	1^o^	0
Lee et al. [53]	2016	Case-control	2006–2014	South Korea	1606		NA	NA	PTB,EPTB	4.5	1^o^,2^o^	39.85
Assefa et al. [54]	2017	Case-control	2013	Ethiopia	710	229	47.6	31.7	PTB	NA	1^o^,2^o^	NA
Workicho et al. [55]	2017	Case-control	2011	Ethiopia	180	90	45.60	29.4	PTB	NA	1^o^,2^o^	91.10
Sinha et al. [56]	2017	Cross-sectional	2012–2014	India	235	124	45.16	35.7	PTB, EPTB	9.68	1^o^,2^o^	25.80
Mesfin et al. [57]	2018	Cross-sectional	2015–2016	Ethiopia	226	89	41.60	34.4	PTB	NA	1^o^,2^o^	82
Gobena et al. [58]	2018	Case-control	2016–2017	Ethiopia	132	59	61	30.2	PTB, EPTB	3.40	1^o^,2^o^	64
Kusumawati et al. [59]	2018	Cross-sectional	2010–2013	Indonesia	842	98	72.40	44.5	PTB	NA	1^o^,2^o^	64.30
Pavlenko et al. [60]	2018	Cross-sectional	2013–2014	Ukraine	1658	474	75	~ 43	PTB	NA	1^o^,2^o^	37.92
Desissa et al. [61]	2018	Case-control	2016	Ethiopia	219	73	45.20	32.69	PTB, EPTB	26	1^o^,2^o^	65.80
Gaborit et al. [62]	2018	Case-control	2002–2013	France	134	44	75	~ 33	PTB, EPTB	36.36	1^o^,2^o^	52.27
Alene et al. [63]	2019	Case-control	2010–2015	Ethiopia	452	242	60.70	~ 31	PTB, EPTB	6.20	1^o^,2^o^	93
Baya et al. [64]	2019	Cross-sectional	2007–2016	Mali	214	134	76.12	39.3	PTB	NA	2^o^	100
Zurcher et al. [65]	2019	Cross-sectional	2013–2016	Cote d’Ivoire, Demo graphic Republic of the Congo, Kenya, Nigeria, South Africa, Peru, Thailand	871	163	60	33.2	PTB	NA	2^o^	100
Okethwangu et al. [66]	2019	Case-control	2013–2017	Uganda	125	33	NA	~ 42	PTB	NA	2^o^	100
Fikre et al. [67]	2019	Case-control	2016–2018	Ethiopia	204	102	67.70	35.6	PTB, EPTB	8.80	1^o^,2^o^	72.50
Elduma et al. [68]	2019	Case-control	2017–2019	Sudan	1290	430	69.77	37.3	PTB	NA	1^o^,2^o^	67.90
Chan et al. [69]	2020	Surveillance	2011–2016	USA	45,209	615	52.2	~ 45	NA	NA	1^o^,2^o^	18.04
Arroyo et al. [70]	2020	Cohort	2006–2016	Brazil	167,726	866	70.7	~ 45	PTB, EPTB	3.4	1^o^,2^o^	54.70
Hirama et al. [71]	2020	Cohort	2010–2016	Canada	402	46	47.8	~ 45	PTB, EPTB	26.08	1^o^,2^o^	34.80

*MDR-TB* Multidrug-resistant tuberculosis, *PTB* Pulmonary tuberculosis, *EPTB* Extra-pulmonary tuberculosis, *PTC* Previously treated cases

### Data extraction

A pre-specified and standardized form was used for data abstraction. For the estimation of the crude odds ratios, key data on the number of MDR-TB HIV positive, number of MDR-TB HIV negative, number of non-MDR-TB HIV positive, and number of non-MDR-TB HIV negative patients were extracted from each study. Additionally, data on the name of the first author, country, year of publication, years of recruitment, study design (cross-sectional, case-control or cohort), source of data (hospital/medical records, lab reports, database/register, or direct from the patients), methods carried out to determine drug resistance pattern, number of enrolled TB patients, the mean or median age of the MDR-TB cases, proportion of male patients among MDR-TB cases (%), type of MDR-TB (primary, secondary or both), form of tuberculosis (pulmonary, both pulmonary and extra-pulmonary or not defined), and proportion of the patients with extra-pulmonary tuberculosis among MDR-TB cases (%) were entered in the spreadsheet. We further stratified the articles based on global regions (defined by World Health Organization) income level (World Bank classification by income, GNI per capita) and burden for MDR-TB (list used by WHO 2016–2020) of the countries [1, 72]. Data abstraction from individual studies was executed by two trios of investigators (AI, JB & ZZS and HRK, DHH & FH), from March 2019 to June 2020. The presence of any inconsistency in data extraction was verified by a seventh investigator (AH).

### Quality assessment

Two trios of investigators (AI, JB & ZZS and HRK, DHH & FH) scored each study to ascertain methodological quality independently. The corresponding author (AH) subsequently examined all the assessments. Furthermore, contentions aroused in the course of quality scoring were resolved through discussions between investigators. It was evaluated using the Newcastle-Ottawa Scale (NOS). It consists of three domains: selection, comparability, and exposure or outcome of interest. Few points on the NOS related to appropriate methods for evaluating exposure variables and outcomes were designed for the relevance to our research question (Supplementary file 1). Two outcome groups were considered comparable if the HIV status was adjusted for the previous history of diagnosis with tuberculosis and/or any other sociodemographic variable (e.g., age, sex, etc.). These studies also reflect high or medium NOS scores (Supplementary file 2*)*. Articles were given scores to reflect methodological stringency, lucidity, and transparency in reporting. Nevertheless, we did not exclude any articles based on quality scoring. It may exclude studies coming from resource-limited settings. Therefore, we did not provide insight into this particular cluster of studies. Moreover, the PRISMA statement consists of a checklist of 27 items and is given in Supplementary file 3.

### Statistical analysis

We performed data analysis using *meta* and *metafor* packages in the R statistical software (version 3.5.1). We calculated crude odds ratio with a 95% confidence interval (CI) for individual study from the abstracted frequencies (numerators and denominators). After that, we estimated the pooled odds ratio (overall) using the random-effects model, allowing the *true effect size* to vary from study to study. The summary estimate (OR) for the association between HIV infection and MDR-TB were reported with 95% CI. The calculated odds ratio of each study and the combined effect estimate with 95% CI were graphically represented by forest plots. Publication bias was assessed by observing the symmetry of funnel plots visually. Further confirmation was conducted using Egger’s test (weighted regression with multiplicative dispersion model), while the *p-value* < 0.10 was suggestive of statistical significance. Heterogeneity across the selected studies was assessed by *I*^2^ statistic (> 75% signifies substantial, 50–75% moderate and 25–50% low heterogeneity). The *I*^2^ statistic represents the percentage of total variation across studies due to heterogeneity rather than chance. We also conducted a sensitivity analysis that removed the study contributing to the highest weight to evaluate the robustness of the findings.

Analysis of the subgroups was carried out to determine the pooled odds ratio for each group and to look for potential explanations of the heterogeneity. Pre-determined subgroups were WHO global regions (Africa, Europe, South-east Asia, America, Pacific, and Eastern Mediterranean), the income level of the country (high, upper-middle, lower-middle and low), the burden for MDR-TB on the country (high and low), design of the study (cross-sectional, case-control and cohort), type of MDR-TB (primary, secondary and both), mean or median age 40 years and older, and diagnostic method used for MDR-TB (culture and culture and/or molecular technique). Funnel plot asymmetry and egger’s tests were done to assess the presence of publication bias in each subgroup.

## Results

**Fig. 1:**
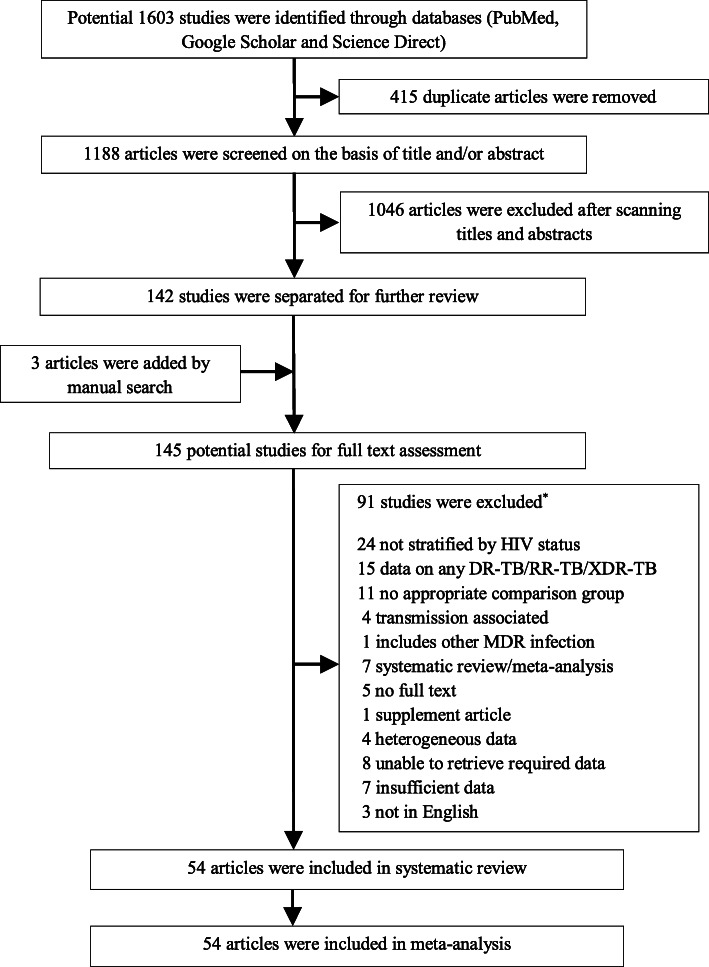
PRISMA flow diagram for Study selection

Our systematic review identified 1603 studies through a database search. After eliminating the duplicates, titles and abstracts of 1188 articles were scanned by the investigators to retrieve a set of relevant studies for further review. We subsequently narrowed down to 145 possible studies, and three were added for full-text evaluation through manual search from the reference lists of included studies. Finally, 54 studies were selected in our systematic review and meta-analysis, including 430,534 TB patients (Fig. 1). The characteristics of the selected studies can be found in Table 1.

(*Studies were excluded because of more than one rationale and the references of excluded studies are given in Supplementary file 4.)

The retrieved articles represented 36 countries from all WHO global regions (Africa, Europe, South-east Asia, America, Pacific, and Eastern Mediterranean). Of the 54 studies, three were conducted in multiple centers in different countries (Table 1). Nearly half of studies were reported from the African region. Many of the studies were performed in Ethiopia and from high MDR-TB burden countries.

The systematic review included 20 case-control studies and 31 studies considering tuberculosis as a pulmonary form. Of the 54 studies, 42 investigated both primary and secondary MDR-TB. The proportion of MDR-TB cases with extra-pulmonary tuberculosis varied from 2 to 36.36%. The proportion of previously treated tuberculosis among multidrug-resistant cases ranged from 12.00 to 99.11% (39 studies). It shows from Table 1 that only culture-confirmed MDR-TB cases were reported in 37 of the studies.

**Fig. 2:**
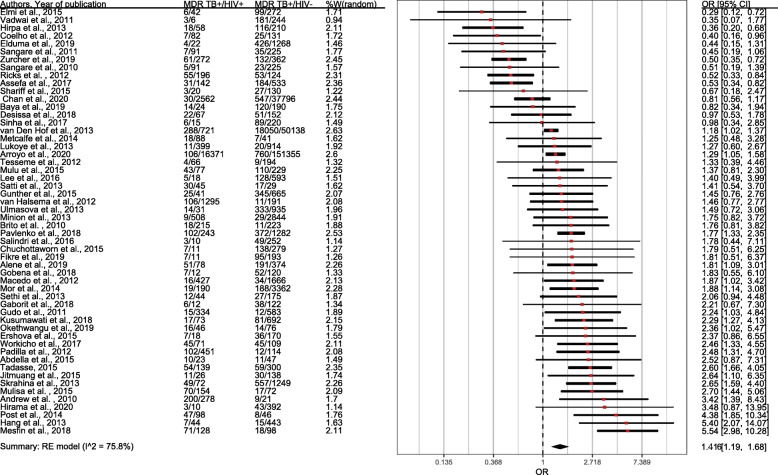
Forest plot exhibiting OR of each study and combined OR of the crude association between HIV infection and MDR-TB

**Fig. 3:**
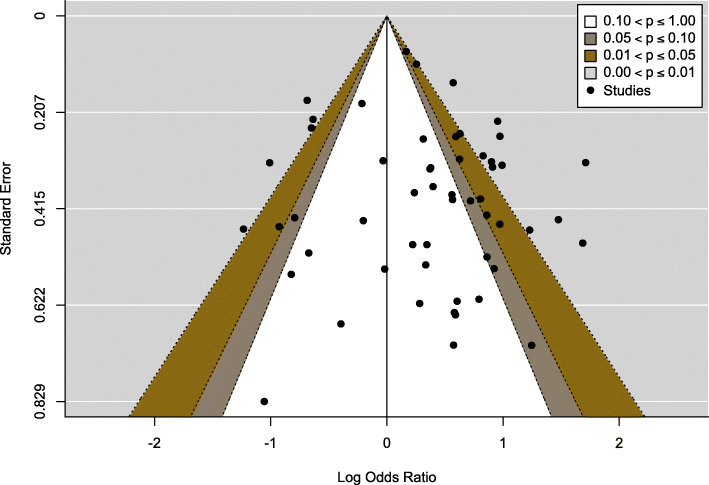
The funnel plot reveals existence of publication bias though most points fall within the 95% confidence region. Each point represents a study; the y-axis represents standard error, and the x-axis displays the ratio of the log odds of the study

It appears from Fig. 2 that the overall pooled odds ratio is 1.42 (95% CI= 1.17–1.71), which suggests that the odds of developing MDR-TB in HIV-infected patients was 42% higher than those of HIV-negative individuals. The evidence of publication bias was tested by visual examination of funnel plot symmetry, and further, the absence of the publication bias was supported by the Egger test (*p*=0.36) and is shown in Fig. 3.

For the South-East Asian countries, the pooled odds of MDR-TB was 1.86 times higher for HIV positives than HIV negative individuals (OR=1.86, 95% CI=1.30–2.67). Most studies obtained from the African region had a pooled odds ratio of 1.41 (95% CI= 1.06–1.89). Results from WHO regions indicate that MDR-TB among HIV-persons is more in South-East Asian countries compared to other regions. The pooled ORs show lower odds of developing MDR-TB among HIV patients in upper-middle-income countries (OR=1.26, 95% CI=0.86–1.86) and low-income countries (OR=1.40, 95% CI=0.99–1.98) compared to high-income countries. The pooled odds ratio in the high TB-burden countries (OR=1.75, 95% CI= 1.39–2.19) was found to be significantly higher compared to the low TB-burden countries (OR=1.0, 95% CI= 0.72–1.39).

Findings from subgroup analysis in Table 2 shows the pooled odds ratio of the cross-sectional studies was 1.53 (95% CI= 1.20–1.96). Cohort studies provides odds ratio of 1.33 (95% CI=1.09–1.62), and case-control studies provides odds ratio of 1.22 (95% CI=0.87–1.71). Cross-sectional studies contribute about 70% of the weight of the overall meta-analysis research. For primary MDR-TB, the estimate was higher than secondary MDR-TB (OR=2.76, 95% CI= 1.70–4.46) with no heterogeneity among the studies. The majority of studies included both primary and secondary MDR-TB (42 studies), and subgroup analysis reveals that the odds of developing both primary and secondary MDR-TB in people infected with HIV were 1.42 times more compared to the individuals without HIV (OR=1.42, 95% CI= 1.16–1.74). Furthermore, the trend towards the development of MDR-TB in HIV-positive people increased with age (i.e., mean or median age of cases 40 years and older, OR=1.56, 95% CI= 1.17–2.06, and for below 40 years of age is OR=1.45, 95% CI= 1.11–1.91). In HIV infected patients, the pooled odds ratio of culturally confirmed MDR-TB cases (OR=1.52, 95% CI=1.20–1.93) was slightly higher than the mixed (i.e., culture and molecular technique) (OR=1.30, 95% CI=0.93–1.81). Only one study (OR=1.27, 95% CI= 0.6–2.17) considered the diagnosis of MDR-TB by molecular technique.

**Table 2:** Summary results of subgroup analysis

Subgroup	No of studies (TB patients)	Summary OR	95% CI	I^**2**^ (%)	***p***-value*
**WHO Region**					
South-East Asia	6 (1947)	1.86	1.30–2.67	0.00	0.07
Europe	11 (154031)	1.79	1.42–2.27	52.90	0.01
Eastern Mediterranean	1 (634)	0.44	0.15–1.31	–	–
Africa	25 (14723)	1.41	1.06–1.89	76.65	0.53
America	6 (256640)	1.17	0.75–1.84	74.15	0.98
Western Pacific	4 (2559)	1.11	0.32–3.89	82.89	0.97
**TB Burden countries**					
Low	18	1.0	0.72–1.39	63.91	0.71
High	30	1.75	1.39–2.19	76.68	0.09
**Countries by income level**					
High	7	1.55	1.06–2.27	50.85	0.09
Upper-middle	14	1.26	0.86–1.86	87.0	0.85
Low-middle	11	1.66	1.23–2.26	43.91	0.31
Low	19	1.40	0.99–1.98	78.44	0.97
**Study design**					
Cross-sectional^a^	30	1.53	1.20–1.96	71.6	0.27
Case-control	20	1.22	0.87–1.71	75.77	0.26
Cohort	4	1.33	1.09–1.62	0.00	0.20
**Outcome MDR-TB type**					
Primary	4	2.76	1.70–4.46	0.00	0.87
Both	42	1.42	1.16–1.74	80.63	0.50
Secondary	8	1.08	0.64–1.82	76.26	0.18
**Mean/Median Age (MDR-TB)**					
> 40	15	1.56	1.17–2.06	71.66	0.57
<=40	32	1.45	1.11–1.91	78.85	0.09
**Diagnosis of MDR-TB by**					
Culture	37	1.52	1.20–1.93	80.56	0.40
Mixed	14	1.30	0.93–1.81	76.97	0.48
Molecular technique	1	1.27	0.6–2.17	–	–
**Overall**	54	1.42	1.17–1.71	75.8	0.36

*MDR-TB* Multidrug resistant tuberculosis, *HIV* Human Immunodeficiency Virus, *CI* Confidence Interval

^a^ two surveillance were included into cross-sectional studies

^*^ *p* value for Egger’s test for publication bias

### Sensitivity analysis

We conducted sensitivity analyses that excluded each of the following types of studies: studies with fewer than 1000 participants; studies from countries in Africa; and studies published in 2015 or earlier. Forest plots are reported in Supplementary file 5. When considering studies of more than 1000 participants, the OR of MDR-TB among HIV infected individuals tends to be 1.41 (OR=1.41, CI=1.13–1.76). The OR is 1.38 (CI=1.01–1.88) considering studies published after 2015. The OR is also 1.46 when studies outside African studies (OR=1.46, CI=1.14–1.87) are considered. The findings are, therefore, similar to the meta-analysis with 54 studies.

## Discussion

About half a million TB patients were included from 54 studies in our systematic review and meta-analysis. Based on the findings of this meta-analysis, the odds of MDR-TB among HIV-positive cases were 1.42 times higher, and this was statistically significant. An earlier pooled study by Mesfin et al. (OR=1.24, 95% CI= 1.04–1.43) included 24 studies published from 1994 to 2011, and our finding appears to indicate an upward trend of odds ratio after being compared to it. Moreover, the 18 cross-sectional studies from Mesfin et al. gave pooled effect estimate for MDR- TB and HIV was 1.26 (OR=1.26, 95% CI= 1.02–1.49) while the result from our meta-analysis (30 studies) is 1.55 (OR=1.55, 95% CI= 1.26–1.95). Another meta-analysis (1988–2007) did not report a pooled effect estimate due to high heterogeneity among the studies [15]. Comparing with Mesfin et al. meta-analysis, it suggests over the last 10 years that people diagnosed with HIV are more likely to have MDR-TB.

Subgroup analysis by the WHO global regions reveals that the OR of South-East Asia was found the highest, followed by Europe and Africa. The WHO South-East Asia region is a home for a quarter of the world ‘s population, with a 44% TB burden, and the second-highest HIV prevalence [1]. Additionally, one-third of the global MDR-TB lies in this region [1]. In Europe, the proportion of MDR-TB among HIV-infected people increased sharply between 2008 and 2017, from 3 to 12%. The aforementioned data ratifies how our results reflect a major risk of MDR-TB among HIV patients.

Furthermore, the estimated pooled OR was found to be significant for the high TB burden countries. In our meta-analysis, with the age of 40 years and older, the pooled odds ratio of developing MDR-TB among HIV infected individuals continues to increase significantly. Findings from the subgroup study also showed that the pooled odds ratio of the cross-sectional studies was higher than that of the cohort studies. Similar findings were also seen in the previous meta-analysis [16].

Another notable finding was the significant association between HIV infection and primary MDR-TB. This finding corresponds to the previous two systematic reviews [15, 16]. In many instances, despite being infected primarily with drug-resistant strains and subsequent development of MDR-TB (primary), it will initially be reported as drug-susceptible tuberculosis. Drug susceptibility test is not routinely done in some settings and performed only after the failure of initial treatment, which will normally be classified as secondary MDR-TB [73]. We found an insignificant pooled odds ratio between HIV and MDR-TB in low-income countries, especially in Africa. It highlights the need to develop effective drug-resistance diagnosis in many resource-limited settings. Among the high-income countries, the combined estimate was found higher than that of the low-income countries. It is important to gain a clear understanding of these mechanisms to build effective strategies to control the expansion of MDR-TB in HIV patients.

In our systematic review, we found most studies considered the diagnosis of MDR-TB as a phenotypic approach that confirmed by culture. The pooled odds ratio of MDR-TB was considerably higher for studies when a diagnosis for MDR-TB was made by culture-confirmed than the mixed or molecular technique. Such culture-confirmed traditional approaches take months to confirm the diagnosis of MDR-TB and ultimately lead to delay in treatment, increased transmission, and poor outcome [74]. However, it should also be noted that the diagnosis of tuberculosis in patients infected with HIV is difficult due to reduced bacterial load and cavitation, as well as poor performance of the standard diagnostic tools [75–77]. Additionally, HIV-associated superinfection may be a motivating factor for drug-susceptible conversion to resistant tuberculosis [78]. Research also shows that multiple tuberculosis strain infections frequently interfere in HIV patients [79, 80]. It may also show misleading phenotypic drug-resistance diagnosis [81]. As such, the extent of spread when confirming MDR-TB by a traditional method is far more deadly than is illustrated, especially in HIV-endemic settings.

We acknowledge that our systematic review and meta-analysis has limitations. There was disproportionate allocation of studies among the six WHO regions. Additionally, the included studies were mostly from Africa (i.e., 25 of 54 studies from Africa). Moreover, the largest nation, China, is not included in the study. Besides, the database search language was restricted to English. Therefore, the above limitations might curb the generalizability of our findings. Moreover, substantial heterogeneity was observed between the studies, although it was expected to be from the differences in MDR-TB and HIV ascertainment, study design, and data collection methods between the selected studies. Mild or no heterogeneity was also observed in a different subgroup analysis. We included only the observational studies which are susceptible to selection bias.

## Conclusion

The meta-analysis clearly shows a growing trend in MDR-TB risk among HIV-infected people. Balanced resource allocation for Asian, European and African countries should be considered to halt both primary and secondary MDR-TB, especially among those with increasing age. As such, the enhancement of the diagnosis and proper overall management of MDR-TB among HIV-positive individuals has become crucial in achieving WHO’s goals of ‘End TB’ by 2035.

## Supplementary Information

**Additional file 1 **: **Table S1.** Study quality assessment details for case-control, cohort, and cross-sectional studies.

**Additional file 2 **: **Table S2.** Quality Assessment of Included Studies by New-Castle Ottawa Scale.

**Additional file 3 **: **Table S3.** Preferred Reporting Items for Systematic Reviews and Meta-analysis (PRISMA) checklist.

**Additional file 4.** References of studies excluded after full-text review.

**Additional file 5.** Sensitivity analysis.

## Data Availability

The datasets used and/or analysed during the current study are available from the corresponding author on reasonable request.

## Appendix

**Table 3:** Search strategy

**A. PubMed**		
#1	“Multidrug resistant tuberculosis” [All Fields] OR “MDR-TB” [All Fields] OR “Drug resistant TB” [All Fields] OR “Risk factors of MDR-TB” [All Fields] OR “Predictors of MDR-TB” [All Fields]	21,717
#2	“HIV” [All Fields] OR “Human Immunodeficiency Virus” [All Fields]	287,526
#3	#1 AND #2	9423
Searching date starting from 01/01/2010 to 30/07/2020		
**B. Google scholar**		
#1	“Multidrug resistant tuberculosis” OR “MDR-TB” OR “Drug resistant TB” OR “Risk factors of MDR-TB” OR “Predictors of MDR-TB”	226,850
#2	“HIV” OR “Human Immunodeficiency Virus”	1,570,000
#3	#1 AND #2	154,780
Searching date starting from 01/01/2010 to 30/07/2020		
**C. Science direct**		
#1	“Multidrug resistant tuberculosis” OR “MDR-TB” OR “Drug resistant TB” OR “Risk factors of MDR-TB” OR “Predictors of MDR-TB”	25,448
#2	“HIV” OR “Human Immunodeficiency Virus”	192,835
#3	#1 AND #2	20,070
Searching date starting from 01/01/2010 to 30/07/2020		

## Abbreviations

MDR-TB: Multidrug-resistant tuberculosis
HIV: Human immunodeficiency virus
WHO: World Health Organization
OR: Odds Ratio
CI: Confidence interval of odds ratio
