# Correction to: HIV infection and multidrug resistant tuberculosis: a systematic review and meta-analysis

**DOI:** 10.1186/s12879-021-05799-0

**Published:** 2021-01-20

**Authors:** Zeeba Zahra Sultana, Farhana Ul Hoque, Joseph Beyene, Md. Akhlak-Ul-Islam, Md Hasinur Rahman Khan, Shakil Ahmed, Delwer Hossain Hawlader, Ahmed Hossain

**Affiliations:** 1 CAPABLE- A Cambridge-led program in Bangladesh, University of Cambridge, Cambridge, UK; 2 Department of Public Health, North South University, Dhaka, Bangladesh; 3 Department of Health Research Methods, Evidence, and Impact, Faculty of Health Sciences, McMaster University, Hamilton, Ontario Canada; 4 Department of Hematology, Bangabandhu Sheikh Mujib Medical University, Dhaka, Bangladesh; 5 Institute of Statistical Research and Training, University of Dhaka, Dhaka, Bangladesh; 6 Global Health Institute, North South University, Dhaka, Bangladesh; 7 Health Management BD Foundation, Dhaka, Bangladesh

**Correction to: BMC Infect Dis 21, 51 (2021)**

**https://doi.org/10.1186/s12879-020-05749-2**

After publication of the original article [1], the authors identified a typo in the Abstract, Methods section: it is written January 12,010, but it should be January 1, 2010.

The original article has been corrected.

## References

[1] Sultana (2021). HIV infection and multidrug resistant tuberculosis: a systematic review and meta-analysis. BMC Infect Dis.

