# Supplementary material for: HIV infection and multidrug resistant tuberculosis: a systematic review and meta-analysis
Source: BMC Infect Dis. 2021 Jan 11;21:51. doi: 10.1186/s12879-020-05749-2 (PMC7802168; doi:10.1186/s12879-020-05749-2)
Supplement: Supplementary file 1 — Additional file 1 : Table S1. Study quality assessment details for case-control, cohort, and cross-sectional studies. [file 12879_2020_5749_MOESM1_ESM.docx]

| **CROSS-SECTIONAL STUDIES** | | | | | | | | |  |
| --- | --- | --- | --- | --- | --- | --- | --- | --- | --- |
| **Ref** | **Selection (Max 5)** | | | | **Comparability (Max 2)**  of case and control by design/analysis | **Outcome (Max 3)** | | **NOC [10]** |  |
|  | Representative-ness | Sample Size | Nonrespondents | Ascertainment of exposure |  | Assessment of outcome | Statistical test |  |  |
| Brito et al. [28] | Six hospitals of Rio De Janeiro were chosen conveniently. | All patients were registered and who had bacteriological confirmation of TB were taken. [Pre-defined eligibility criteria.] Justified. ★ | 10.20% were not interviewed or didn’t give consent. ★ | Patients were tested for HIV by enzyme-linked immunosorbent assay and Western blot or immune-fluorescence as a confirmatory test. ★★ | Adjusted for: age, marital status, basic sanitation, alcohol abuse, TB reference hospital, previous admission to TB hospital, **previous TB treatment**, **HIV status**, cough, chest X-ray and fever★★ | DST done by the proportion method using LJ medium. Laboratory technicians were blinded to chest radiograph results and clinical predictors. ★★ | Described, association with CI and p value were included. ★ | **9** |  |
| Sangare et al. [30] | National Tuberculosis (TB) Control Centre and two non-specific TB center. Which method it was selected was not mentioned. | Prevalence of resistance at 10%, precision at 4%, the cluster effect 2, risk α = 5% and losses 10%. In PTC, we used 100% sampling. Total sample size of both types was therefore 538; rounded it to 550 PTB cases. ★ | 416 (75.6%) were eligible and were consecutively included from two specific TB reference centers. Specific causes are described. Justified. ★ | HIV status was determined according to the Joint United Nations Programme on HIV/AIDS/WHO recommendations. ★ | No adjustment was done for HIV. | Culture (LJ media) and DST by proportion method. External quality control was performed at the Borstel Mycobacteria Reference Centre, Borstel, Germany. ★★ | Adequately descriptives given. Association was measured by chi-square and p-value was provided. ★ | **6** |  |
| Sangare et al. [31] | 2 great TB hospital and other two centers were chosen purposively where representative case can be found. ★ | Patients who accepted to be tested for HIV antibodies were included. | Non-response rate was not ascertained. | Two rapid tests were performed (UNAIDS/WHO). Determine HIV1-2 and afterwards with ImmunoComb II Bispot HIV 1&2. ★★ | No adjustment was done. | Culture (LJ media) and DST (by proportion method). Tested at CNLAT centers in collaboration with National Reference Center for Mycobacteria in Borstel. ★★ | Descriptive and p-value only. No clear measure of association shown, not even CI. | **5** |  |
| Gudo et al. [32] | Designed according to WHO, 40 diagnostic center in 11 province. ★ | Sampling was probability proportional to size, based on number of new sputum smear +ve cases. Each cluster to enroll 30 new sputum. Justified. ★ | 1398 sputum were taken, where 80.6% could be adequately recovered, sufficient according to WHO. ★ | Structured questionnaire followed by cross-checked by medical record. ★ | No adjustment was done. | Culture and DST in LJ media at the National Reference Laboratory. 10% sample were randomly selected and were sent to Supranational Reference laboratory in Italy for cross-check. ★★ | No description. | **6** |  |
| Vadwai et al. [33] | CAP and RNTCP was selected with a reference bias towards treatment failure. | All patients who have phenotypic results available. | 64.76% with detailed histories were selected for study. | Retrospective data from patient record. ★ | No adjustment was done. | DST was done by MGIT SIRE (Becton Dickinson, NJ, USA) ★ | Described, association with CI were included. ★ | **3** |  |
| Macedo et al. [34] | All TB cases from the TB surveillance data (SVIG-TB). ★ | All culture-confirmed TB cases in LVT in 3 year period. Justified. ★ | All respondents were included in the analysis. ★ | Standardized form by physician with records. ★ | No adjustment was done. | Culture followed by DST (liquid medium, BACTEC MGIT 960) was performed by quality assured laboratory. ★★ | Descriptives with association measure with CI and p-value ★ | **7** |  |
| Padilla et al. [35] | Based on WHO guidelines for surveillance of drug resistance TB, including 15 TB diagnosis centers of 4 region of Swaziland. 988 were screened and 840 met eligibility criteria★ | Prevalence of RR was used, 5% for NC and 15% for PTC. Absolute error 2.5% for NC and 4.0% for PTC, and 95% CI. 20% non-response rate was added. ★ | 15.9% were not screened due to heavy workload and staff turnover. Further, 4.05% were not included because they didn’t gave consent or couldn’t produce sputum. ★ | According to national guideline, +ve results for 2 rapid HIV tests were taken for declaring HIV positive. If any discordance between 2 tests, DNA PCR using dried blood spot performed at national ref lab. ★★ | Adjusted for in multivariate analysis was age, sex, **HIV status** and **TB patient type (new case and previous treated)**, after they were significant in univariate analysis (p value 0.05). ★★ | Objective validated laboratory, Culture followed by DST in Indirect proportion method on LJ media.★★ | Descriptives with association measure with CI and p-value ★ | **10** |  |
| vanHalsema et al. [36] | Gold-mining worforce population. Data from two mining companies from Gauteng Province, South Africa. Somewhat represent. Purposive selection. ★ | All TB episodes with culture confirmation were included if the treatment started between 2002 and 2008. Justified. ★ | Non-respondent rate were described adequately. And it was >80% in every calculation. ★ | HIV status was defined by positive rapid test or enzyme-linked immunosorbent assay result. +ve before anti-TB treatment, negative even after 6 month treatment. ★★ | No adjustment was done. | Culture and DST using BACTEC MGIT 960 system (BD, Sparks, MD, USA) in the laboratories serving the mine health services. ★★ | Descriptives with association measure with CI and p-value ★ | **8** |  |
| Tesseme et al. [38] | Gondar Hospital, the Gondar Health Centre, the Metemma Hospital, the Bahir Dar Hospital and the Debre Markos Hospital. How it was selected was not mentioned. | No sample calculation was provided. Clearly not stated whether all patients were taken. | Non-response rate was not ascertained. | Serum samples were screened for HIV-1 and HIV-2 using the Vironostika HIV Uni-Form II Ag/Ab enzyme-linked immunosorbent assay (ELISA) kit. ★★ | No adjustment for any variable. | Specimens were processed and cultured according to the DIN recommendations using LJ media, Gottsacker media and BacT/ALERT 3D system (BioMerieux, Marcy l’Etoile, France). ★★ | Descriptives with association measure with CI and p-value ★ | **5** |  |
| Coelho et al. [39] | Study population consists of patients who were diagnosed and treated for pulmonary TB living in Santos. ★ | All patients from the TB surveillance system meeting the eligibility criteria were included. ★ | The number not responded to underwent DST, even it was indicated, 68.27%. | TB surveillance system, Santos Regional library and Central Laboratory, either. ★ | No adjustment was done. | Culture and DST using resistance ratio method in the laboratories previously mentioned, under National TB programme. ★★ | Not adequately mentioned and done. | **5** |  |
| Ulmasova et al. [40] | Sampling frame consisted of pulmonary TB patients diagnosed at public healthcare facilities in all administrative regions of Uzbekistan. ★ | Expected prevalence: 15%, Absolute error: 2.5%, 95% CI. 818 cases required inclusive of 20% margin to cover for expected losses. 12 months with 100% sampling ★ | More than the calculated sample was included in the study. ★ | All patients were offered HIV testing and were counselled.★ | Not adjusted for HIV infection status. | Processed in both LJ media and MGIT media. DST was performed using method proportion and MGIT. Positive culture were then tested using Genotype MTBDR plus for the detection of resistance-conferring mutation. If no mutation was identified, SRL repeated DST in liquid media and then considered definitive. ★★ | Clearly described with CI, adequately done. ★ | **7** |  |
| Minion et al. [41] | All cases of 12 year period was included which is monitored by Public Health Agency of Canada (Reporting and surveillance system) ★ | All patient with culture confirmed TB were included meeting the criteria. ★ | Information could be retrieved. Non-respondents in each exposure were included in analysis. ★ | Abstracted from the database information (case and lab info). ★ | Not adjusted for any confounder. | Canadian laboratories performed routine susceptibility testing of MTB complex isolates using either BACTEC 460 or MGIT 960. All laboratories participates in proficiency testing programs conducted by the NRCM. ★★ | Described adequately with associations expressed with CI. ★ | **7** |  |
| Sethi et al. [42] | Suspected TB coming to a tertiary chest hospital in Chandigarh, India. Might not represent the population. | Purposively based on eligibility criteria, after confirmation of TB. Justified ★ | All patients which was classified as MDR-TB were included. ★ | HIV screening was according to NACO guidelines. ★★ | Association of MDR-TB with **HIV status** was established after controlling age, sex and **previous treatment status** by multivariable regression analysis. ★★ | Tests are conducted at PGIMER at the Revised National TB Control Programme, testing accredited by Central TB Division. Culture followed by DST using standard proportion method. ★★ | Described, association with CI and p value were included. ★ | **9** |  |
| van Den Hof et al. [43] | Routine data from electronic national TB register of Kazakhstan includes all TB cases (2007-2011). ★ | All notified and started on treatment TB cases were included in the analysis. Justified. ★ | All patients with HIV and DST results available. Response rate 36%. | Surveillance system. ★ | The multivariate model includes adjustment for year, age, **HIV status**, **patient category (new or old case treated)**, homeless and history of incarceration. ★★ | Cultured at LJ media and DST using absolute concentration method. Tests done by national reference laboratory for TB and quality assurance by Supranational Laboratory in Germany. ★★ | Described adequately with associations expressed with CI. ★ | **8** |  |
| Lukoye et al. [44] | A cluster sampling method was used in which 30 clusters, selected randomly with probability proportional to the number of smear +ve TB patients registered. ★ | RR-TB prevalence: 1.4%, absolute precision: 1%, 95% CI, design effect 2, estimated loss 15%, final sample 1500, 50 in each cluster within a year. ★ | 14.7% data were not included in the final sample (corresponds to estimated loss due to contamination & -ve culture) ★ | Data were collected by structured questionnaire. All TB patient eligible for enrollment were counselled and tested for HIV as required by Uganda NTLP ★ | Not adjusted for the exposure of HIV. | DST was done by L-J proportional method. Blinded external quality assurance was also done for random sample by supra-national reference laboratory. ★★ | Described, association presented with CI. Appropriate.★ | **7** |  |
| Hang et al. [45] | 7 of 14 districts were selected, >50% new smear +ve TB were diagnosed and treated in the area. Selection method was not described. | No calculation provided. | Recruited based on eligibility criteria from local TB care unit. 10.44% respondent’s data were not available. ★ | Blood samples were obtained for HIV testing and records kept for registration with the national TB program in district TB center. ★ | Adjusted for MTB strain type to see the association between MDR-TB and **HIV status**. ★ | Culture on LJ media, DST according to WHO standard proportional method. ★★ | Described, association with CI and p value (bold) were included. ★ | **6** |  |
| Skrahina et al. [46] | Sampling frame consists of patients who had pulmonary, smear +ve TB in any of the 196 health-care facilities in Belarus.★ | Described separately for new and previous TB treatment cases. ★ | 95.5% were included in the study. ★ | Oblast-level HIV laboratories and found negative; ELISA-HIV 1, 2-AT, an enzyme immunoassay was used for HIV screening and immunoblot assay was used to confirm the positive results of the screening. ★★ | Adjusted for age, sex, **treatment history**, household no, employment status, history of imprisonment, alcohol, smoking and **HIV status**. ★★ | BACTEC MGIT 960 and/or LJ atone of the eight TB lab. Every successful isolate was investigated at the national TB reference laboratory by BACTEC MGIT 960. ★★ | Described, association with CI and p value were included. ★ | **10** |  |
| Mor et al. [49] | Retrospective study included all diagnosed cases with TB in Israel between 1999 and 2010. ★ | Purposively included all diagnosed TB patients. Justified. ★ | All participants who were included, information could be retrieved. ★ | Data were collected from standard public health database reporting system. ★ | Adjusted for age, sex, country of origin, years of stay in Israel for foreigners, site of infection, **HIV status** and sputum smear. ★ | All culture processed in Israel were sent to National TB Laboratory. Culture on L-J or BACTECT. Followed by DST using resistance ratio method. ★★ | Described, association with CI and p value (bold) were included. ★ | **8** |  |
| Post et al. [50] | Patients chosen from 5 countries from the Large cohort of TB/HIV study. How the country or hospital were chosen. | All patients from the hospitals selected from the countries were selected. ★ | All participant that were eligible and included in cohort, information could be retrieved. ★ | From TB/HIV study primary data. Tests were done for each patient. ★ | No adjustment done. | Culture and DST (LJ media and proportional method. ★★ | Described, association: p value were included only. | **5** |  |
| Metcalfe et al. [51] | Two infectious disease hospitals and suburb of south were chosen and other were not chosen due to lack of logistics. Might not represent. | Patients were consecutively taken with presumptive drug resistant patients from the study location based on eligibility criteria. Justified. ★ | Patients who met the eligibility criteria were included in the study. ★ | Not mentioned. | All patients were previously treated. Groups are comparable in this point. ★ | Culture (LJ media and MGIT) followed by DST (absolute concentration method) with external quality assured laboratory. Includes MODS procedure. ★★ | Described, association with p value were included only. | **5** |  |
| Ershova et al. [59] | Data from a study conducted at the Vladamir Regional TB dispensary, a referral center for TB patients (>25%) and only facility that performs DST in that region. ★ | Sample size or sampling method was not described adequately. | From the presumptive cases, after DST, negative results were declared as Non-MDR. Same source population. (blinded) ★ | HIV was tested for the purpose of study (primary) ★ | No adjustment was done. | Culture and DST (LJ media and MGIT) and GeneXpert positive results. ★★ | Described, association with CI and p value (bold) were included. ★ | **6** |  |
| Abdella et al. [60] | All MDR-TB cases referred from all health facilities in Jimma area to the MRC were included in the study. Selected as this a referral center for MTB culture of Jimma. ★ | All consecutive smears positive pulmonary tuberculosis re-treatment cases. Sample calculation provided and with 20% non-response rate, it was 84 total. ★ | Patients were consecutively taken. Non-respondents were not described. Who didn’t gave consent were not included. | Patient’s clinical record. ★ | Adjusted for **HIV status**, retreatment categories, alcohol abuse and history of being in prison. **All patients were previously treated.** ★ | Cultured in liquid media (BACTEC MGIT 90) and DST by (using BACTEC MGIT indirect proportion method) All done at MRC mycobacteriology laboratory. ★★ | Described, association with CI and p value were included. ★ | **7** |  |
| Tadasse [61] | 26 public health centers in Addis Ababa. Cases selected by SRS at each health center for MDR-TB and lottery and systematic sampling on wards for non-MDR-TB. ★ | Prevalence of re-treatment cases 12%, primary 2%, error 3%, power 80%, 95% CI, 10% were added to cater for incomplete data, total 439. ★ | All patient were included (439). ★ | Secondary data for HIV status: Registry of national TB control program, patient cards, charts. ★ | Adjusted for sex, residence, age, **previous TB treatment**, category of TB, site and **HIV test**. ★★ | Culture and DST but the method were not described. | Described, association with CI and p value were included. ★ | **7** |  |
| Sinha et al. [66] | Tertiary care hospital but why or how the hospital was selected is not described. | Samples from all suspected patient from two referral hospital were taken.Justified. ★ | 721 patients were attained along with information on age and gender. ★ | Documented as examined. Further information was not given. | No adjustment were done for any confounder. | Culture followed by DST was done by conventional 1% proportion method. ★★ | Described and Odds ratio are calculated with CI and P-value. ★ | **5** |  |
| Mesfin et al. [67] | All health facilities which provides laboratory services for MDR-TB diagnosis were selected. Sample analysis was done by EPHI.★ | Prevalence: 17.8%, precision: 5%, CI 95%, z value 1.96, 10% non-response rate, sample size 248 MDR-TB suspected case. ★ | 358 suspected were included, among them 226 had DST results and all were included. ★ | Not described how it was ascertained. | Adjusted for age, smoking, alcohol, **HIV status**, antibiotic treatment history, **TB treatment history**, previously TB infected family member, health facility visit and hospital admitted. ★★ | Gene Xpert MTB/RIF assay and phenotypic DST (BACTEC MGIT 960). As few laboratories do it and others refer all to those, thus those sites were purposively selected. ★★ | Described, association with CI and p value (bold) were included. ★ | **8** |  |
| Kusumawati et al. [69] | TB provincial lab runs all culture & culture based DST, involved in external & internal quality assurance programme. ★ | All patients with culture proven pulmonary TB, performed DST to 1^st^ line anti-TB drugs. Justified. ★ | Information could be availed who were eligible to include in the study. 3.36% DST were not available.★ | HIV status was assessed from the TB registry. ★ | Not adjusted for HIV exposure. | Culture was done by solid LJ medium and DST to first line was performed using the WHO standard conventional proportion method. ★★ | Described, association with CI and p value were included. ★ | **7** |  |
| Pavlenko et al. [70] | Implemented in 40 clusters throughout the country selected using a probability proportional-to-size approach according to notification of new smear-positive TB patients. ★ | All consecutive sputum smear-positive pulmonary TB patients with first line DST results were enrolled. (1550) Justified. ★ | 1550 patient were included in the analysis. Response rate >88%. ★ | Structured questionnaire-cased interview was done for all variable. Medical records were reviewed when available. HIV were offered and tested where unknown with initial and confirmatory test. ★ | Adjusted for age, sex, place of residence, geographic zone of Ukraine, **HIV status** and **previous treatment status** (new or treated). ★★ | DST done by LJ proportional method and was repeated for internal quality assurance by supranational reference laboratory. Resistance conferring mutations were identified in the *pncA* gene. ★★ | Described, association with CI and p value were included. ★ | **9** |  |
| Baya et al. [74] | All presumed TB cases meeting all eligibility criteria coming to national reference unit for MDR-TB tuberculosis. How it was selected not described. | Included all cases meeting eligibility criteria. Patient with incomplete information and no DST were excluded. ★ | 15.41% had incomplete/missing data ★ | Blood samples were taken. Serum was used to test HIV antibodies using Determine HIV ½ Rapid test and HIV Elisa and confirm by western blot. ★★ | Adjusted for age, sex, marital status, **HIV positive**, number of TB treatment, previous treatment failure, smoking, alcohol consumption, close contact with TB patient, bacilloscopy, history of treatment interruption, impaired physical condition and hemoptysis. A**ll patient were previously treated.**★ ★ | Objective validated laboratory method. Inoculation was both done in MGIT and Middlebrook followed by DST on pre-prepared media. Confirmed RR was done by GeneXpert.★★ | Descriptions and association given clearly with CI and p-value ★ | **9** |  |
| Zurcher et al. [75] | International epidemiology Databases to Evaluate AIDS (IeDEA) was used. A global consortium for ART programs with seven countries that are high burden. MDR-TB cross classified by HIV status, 100 patients per site.★ | Sample size was calculated so that the study had adequate power to detect differences in the prevalence of drug resistance between HIV positive and negative. ★ | Not described. | IeDEA database. ★ | All patients included were treated for active pulmonary tuberculosis. ★ | Culture (solid or liquid culture) or molecular technique (adequately described techniques). ★★ | No association was described. No CI or p value. | **6** |  |
| Chen et al. [81] | 2011–2016 surveillance data from the US National Tuberculosis Surveillance System and National Tuberculosis Genotyping Service. ★ | Participants from the surveillance data were included in the study. ★ | All US TB data were included in the study for analysis. ★ | 2011–2016 US surveillance data from the National Tuberculosis Surveillance System (NTSS) database ★ | HIV status was not included in the adjustment model. | Cases were defined by initial DST. But detailed method were not described. ★ | Association was described with confidence interval and p-value. ★ | **6** |  |

| **CASE-CONTROL STUDIES** | | | | | | | | | | | | | | | |
| --- | --- | --- | --- | --- | --- | --- | --- | --- | --- | --- | --- | --- | --- | --- | --- |
| **Ref** | **Selection (Max 4)** | | | | | | | | **Comparability (Max 2)**  of case and control by design/analysis | | **Exposure (Max 3)** | | | | **NOS [9]** |
|  | Adequate case definition | | Representative ness of the cases | | Selection of controls | | Definition of controls | |  |  | Ascertainment of exposure | | Similar for c & c | Non-response rate |  |
| Andrew et al. [29] | Culture (both liquid and solid) followed by DST (1% proportional method, resistant to RH)★ | | All patients diagnosed at COSH with complete medical records. Non-responders were not mentioned. (who doesn’t have complete record) | | During same time consecutively as cases listed in TB DOTS office register in the same hospital. ★ | | Susceptible to all first line drugs ★ | | Hospital admission >14days in past year, **HIV infection**, **previously treated or cured, previously treated or defaulted,** previously treated and failed treatment. Additionally all models were adjusted for age and sex. ★★ | | Hospital record, TB dots clinical record, HIV clinic record ★ | | Yes ★ | Case: 20.23%  Control: 22.41%  Overall: 20.81% ★ | **8** |
| Ricks et al. [37] | Culture and DST but with no internal validation of the methods used. | | No calculation was provided. | | Hospital or respective local community-based DOTS clinic. Thus case and control comes from same population. ★ | | Laboratory confirmed drug susceptible TB were treated with WHO CAT I or II treatment regimens. | | **HIV status** was adjusted for current hospitalization status, **previous TB treatment**, previously hospitalized, household MDR-TB contact, documented HIV testing, receiving ART. ★★ | | Medical data were abstracted from TB treatment cards, patient hospital and TB clinic charts and patient health passports which contain person’s medical history (not blinded). ★ | | Yes ★ | Case: 8%  Control: 16%  Overall: 13.04% ★ | **6** |
| Hirpa et al. [48] | MDR-TB detection was ascertained by LPA or culture (solid media) or DST at EHNRI. ★ | | 147 were eligible during the study period and 8.84% didn’t give consent for the study. ★ | | From Health facility (different population) | | Cured/treatment completed according to WHO criteria★ | | **HIV status** was adjusted for all variable included in the study including **previous TB treatment**. ★★ | | Structured interview, TB and MDR-TB register, controls identified by FMOH screening tool ★ | | Yes ★ | Case: 0%  Control: 0%  Overall: 0% ★ | **8** |
| Shariff et al. [52] | Clinically, radio logically and/or bacteriologically diagnosed as pulmonary TB and MDR by DST ★ | | Purposively selected but no data on which method they used to include cases among the patients. No sample calculation also. | | Hospital (same hospital as cases) ★ | | Same criteria as cases but drug sensitive by DST ★ | | Not adjusted for the exposure of HIV. | | Patient’s medical record. ★ | | Yes ★ | Case: 0%  Control: 0%  Overall: 0% ★ | **6** |
| Jitmuang et al. [53] | Culture by LJ media and/or liquid media system followed by AST (proportion method and/or growth ratio broth): resistant to RH ★ | | All cases of the hospital were included★ | | Hospital control from same source hospital charts (by random sampling). ★ | | Similar to case: not resistant to RH ★ | | **HIV status** was adjusted for age, gender, **previous TB infection**, alcohol consumption and positive AFB smear. ★★ | | Chart review of hospital ★ | | Yes ★ | Case: 12.77%  Control: 12.77%  Overall: 12.77% ★ | **9** |
| Chuchottaworn et al. [54] | DST (LJ media) results from one lab CCIT (central lab) were only taken into consideration with WHO recommendation. ★ | | Consecutive sampling done with strict eligibility criteria. ★ | | From hospital record unit (clinical control from clinical same source) ★ | | Sensitive to all anti-TB drug, matched individually by age, gender & time to time to start anti-TB drug. ★ | | Age, gender & time to time to start anti-TB drug between all case and control. ★ | | Medical Record for both case and control, when not available next control was taken. ★ | | Yes ★ | Case: 0%  Control: 0%  Overall: 0% ★ | **8** |
| Elmi et al. [55] | DST from National reference laboratory which tests all isolates in Malaysia. Cross-checked with MDR-TB registry in TBIS. Method not given. | | Non- probability technique, attended/admitted to selected hospital. How they selected the hospital was not mentioned. | | SRS was applied to select control from clinical source population. ★ | | Fully sensitive to all anti-TB drug ★ | | Model adjusted for immigrant, **HIV infection**, **previous TB treatment** and bacterial load by AFB smear. ★★ | | Patient’s clinical record. ★ | | Yes ★ | Case: 0%  Control: 0%  Overall: 0% ★ | **7** |
| Mulisa et al. [56] | Culture (LJ media) followed by Xpert MTB/RIF or LPA ★ | | All cases of suspected MDR-TB were included and diagnosed MDR-TB were included in the study. ★ | | Different zones of Oromia region ★ | | Following lab assessment negative from the suspected MDR-TB cases.★ | | Adjusted for occupation, education level, TB contact, alcohol use, chronic antacid use, **HIV infection**, and **previous TB history** and treatment outcome. ★★ | | No description. | | No given. | Case: 0%  Control: 0%  Overall: 0% ★ | **7** |
| Mulu et al. [57] | DST were done by Molecular line probe assay ★ | | Purposive selection. The two hospitals that were chosen is the only MDR-TB initiation center. ★ | | Presumptive MDR-TB with negative result.★ | | Sensitive to four drugs/ one resistance in LPA ★ | | Not adjusted for the HIV status exposure. | | Structured questionnaire along with patient’s clinical record review. (blind to case and control status) ★ | | Yes ★ | Case: 0%  Control: 0%  Overall: 0% ★ | **7** |
| Gunther et al. [58] | Culture followed by DST, specific nucleic acid amplification when possible. Quality control through WHO supranational reference lab. ★ | | Consecutive inclusion prospectively at each TBNET sites. ★ | | TBNET site, same cite as cases ★ | | Culture followed by DST, specific nucleic acid amplification when possible, sensitive to drugs.★ | | **HIV status** was adjusted for age, sex, foreign born, imprisonment, injectable drug user and **previous TB treatment**. ★★ | | Not mentioned clearly. | | Not given. | Case: 10.63%  Control: 2.63%  Overall: 6.61% ★ | **7** |
| Lee et al. [63] | Culture positive with antimicrobial susceptibility testing report patients were included. No validation. | | All patients who visited Pusan National University Hospital from Jan 2006 to Oct 2014 were enrolled. ★ | | Same hospital control (same population). ★ | | Comparison were done between HIV positive and negative. | | Not adjusted for any variable. | | Microbiological database. ★ | | Yes ★ | Case: 0%  Control: 0%  Overall: 0% ★ | **5** |
| Assefa et al. [64] | All culture confirmed MDR-TB. No detailed method was not mentioned. | | From all location of Addis Ababa and registered at St. Peter and ALERT ★ | | Health facility, different from where the cases were selected. | | After anti-TB cured/treatment completed ★ | | Educational status, known TB contact history, **previous TB treatment history**, history of hospitalization, smear result at the time of diagnosis, **HIV status**, and diabetes. ★★ | | Structured questionnaire (not blinded) and MDR-TB and TB register. ★ | | Yes ★ | Case: 3.5%  Control: 4.4%  Overall: 4.08% ★ | **7** |
| Workicho et al. [65] | Culture and DST from record | | Consecutively included until the sample size is achieved. Also St peter hospital is the only one hospital where MDR-TB is provided. ★ | | From the same clinical population from where the cases were taken. ★ | | Sputum smear-negative after 2^nd^, 5^th^ or 7^th^ of the treatment course. ★ | | Adjusted for age, number of rooms in the house, **history of previous treatment** and **HIV infection**. ★★ | | Interview and clinical record (data from cases and controls on separate occasion) ★ | | Yes ★ | Case: 0%  Control: 0%  Overall: 0% ★ | **8** |
| Gobena et al. [68] | Not described in which method the cases were selected. | | Sample size was calculated. SRS was used to include participants. ★ | | From the same zone population from where the cases were taken. ★ | | Previous TB patient but declared cure, completed treatment or smear - ve. ★ | | Not adjusted for HIV status. | | Investigator administered questionnaire and TB and MDR-TB register. ★ | | Yes ★ | Case: 0%  Control: 0%  Overall: 0% ★ | **6** |
| Dessisa et al. [71] | Culture and sensitivity testing, no validation how it was assessed. | | Not stated how they select cases from Adama and Bishoftu hospital, even sample. | | TB clinic patient registration book of the same hospital. ★ | | Smear –ve microscopy in intensive or continuation phase. ★ | | Not adjusted for HIV status. | | Structured interview and clinical record. ★ | | Yes★ | Case: 0%  Control: 0%  Overall: 0% ★ | **5** |
| Gaborit et al. [72] | Culture confirmed, then national reference center for phenotypic & genotypic analysis no validation how it was assessed (method). | | Systematic review of medical charts. But the sampling technique or all patients, neither were included. | | Hospital control from the same population where the cases were selected. ★ | | Drug susceptible-TB age, sex and year of diagnosis matched ★ | | HIV adjusted for living in an at-risk area, recent immigration, precarious life, homelessness, incarceration history, professional history, need of an entrepreneur, drug abuse, contact with TB patients, **tuberculosis history**, HCV, HBV, renal failure, cirrhosis and other immune deficiency. Age and sex matched. ★★ | | Interview with specific questionnaire and systematic medical charts review. ★ | | Yes ★ | Case: 0%  Control: 0%  Overall: 0% ★ | **7** |
| Alene et al. [73] | Cuture, DST and Gene Xpert MTB/RIF. No method was described, no validation. | | Cases were all MDR‐TB patients who were confirmed by culture and DST, enrolled at the MDR‐TB treatment centre, from September 2010 to August 2015. ★ | | Hospital control from the same clinical population where the cases were selected. ★ | | Controls were all DS‐TB patients who were confirmed by Gene Xpert MTB/RIF ★ | | Model included age, gender, residence, marital status, educational status, occupation, BMI, **HIV status** and **previous tuberculosis treatment.** ★★ | | Medical and laboratory record. ★ | | Yes ★ | Case: 0%  Control: 0%  Overall: 0% ★ | **8** |
| Okethwangu et al. [76] | Culture, LPA or any other DST. No internal validation about methods. | | How many the patients didn’t have the information, which and why were not described. | | Same record as the cases, SRS used to select 3 control for every case, matched by sub-county of residence. ★ | | On anti-TB medication for at least 2 months and negative for Gene Xpert★ | | Adjusted for adhered to first line TB treatment, **HIV status** and mean symptomatic duration (months) before treatment. **All patients were previously treated** (secondary). ★ | | Hypothesis generating interview. | | No | Case: 0%  Control: 0%  Overall: 0% ★ | **4** |
| Fikre et al. [77] | Culture or LPA. Specific method was not described. | | Sample was adequately calculated. Considering 10% non-response rate, sample was 208. Random selection according to proportion size of the hospitals. ★ | | Same clinical population from which the cases were selected. ★ | | Susceptible to the first line drugs or registered as cured/treatment completed. ★ | | Not adjusted for HIV variable. | | Structured interview and register of TB clinic. ★ | | Yes ★ | Case: 0%  Control: 0%  Overall: 0% ★ | **6** |
| Elduma et al. [78] | Culture and molecular technique was used to identify cases. No specific method was mentioned. | | Sample size calculation was provided. ★ | | Same clinical population from which the cases were selected. ★ | | Susceptible to the first line drugs or registered as cured/treatment completed. ★ | | Not adjusted for HIV variable. | | Structured questionnaire (face to face interview) and report for cases and register for control. | | No | Case: 0%  Control: 0%  Overall: 0% ★ | **4** |
| **COHORT STUDIES** | | | | | | | | | | | | | | | |
| **Ref** | **Selection (Max 4)** | | | | | | | **Comparability (Max 2)**  of case and control by design/analysis  Representativeness of cohort | | **Outcome (max 3)** | | | | | **NOS** |
|  | Representativeness of cohort | Selection of non-exposed cohort | | Ascertainment of exposure | | Definition of controls | |  |  | Ascertainment of outcome | | Ascertainment of exposure | | Adequacy of follow-up |  |
| Satti et al. [47] | All consecutive patients who were registered in the National MDR-TB program were retrospectively included. Representative cohort. ★ | From the same population. ★ | | At the baseline of the study, HIV was ascertained from the medical record. ★ | | From the beginning outcome of interest was accounted. ★ | | All the patients were treated for tuberculosis. ★ | | Outcome ascertained by DST. ★ | | Not adequate. | | <10% were not included. ★ | **7** |
| Salindri et al. [62] | On the basis of eligibility criteria, all the patients were selected. ★ | From the same population. ★ | | Ascertained from record. ★ | | Comparison group who doesn’t develop one of the primary outcome, Primary MDR-TB. ★ | | Adjusted at baseline for age, sex, household income, smoking status, alcohol users, diabetes, **HIV status**, kidney disease. ★ | | TB drug resistance profile by culture and DST (LJ media). ★ | | Not mentioned. | | Follow up or RR was not ascertained by the HIV and MDR-TB. | **6** |
| Hirama et al. [80] | All patients who were diagnosed with TB and treated at WPHC between January 2010 and December 2016 were included in this retrospective cohort study. ★ | From the same population. ★ | | Ascertained from chart review. ★ | | Comparison group who doesn’t have the outcome MDR-TB. ★ | | HIV status was adjusted for history of **previous tuberculosis treatment** and other **sociodemographic variables.** ★★ | | Culture and DST (BACTEC and MGIT90). ★ | | Not mentioned. | | All included who were accounted for. ★ | **8** |
| Arroyo et al. [79] | 645 municipalities in Sao Paulo state, one of the 27 federal units in Brazil. ★ | From the same population. ★ | | Tuberculosis Patient Control System (TB-WEB) ★ | | Comparison group who doesn’t have the outcome MDR-TB. ★ | | HIV status was not adjusted for other variables, where the outcome was MDR-TB. | | Outcome ascertained by culture and/or molecular technique. ★ | | Not mentioned. | | Not mentioned. | **5** |
