# Supplementary material for: HIV infection and multidrug resistant tuberculosis: a systematic review and meta-analysis
Source: BMC Infect Dis. 2021 Jan 11;21:51. doi: 10.1186/s12879-020-05749-2 (PMC7802168; doi:10.1186/s12879-020-05749-2)
Supplement: Supplementary file 2 — Additional file 2 : Table S2. Quality Assessment of Included Studies by New-Castle Ottawa Scale. [file 12879_2020_5749_MOESM2_ESM.docx]

**Supplementary File 1**

**Quality Assessment of Included Studies by New-Castle Ottawa Scale (Adapted)**

| **Case-control and Cohort study** | | | | |
| --- | --- | --- | --- | --- |
| Study | Selection | Comparability | Exposure/Outcome | Score |
| Andrew et. al [29] | ★★★ | ★★ | ★★★ | 8 |
| Ricks et al. [37] | ★ | ★★ | ★★★ | 6 |
| Satti et al. [47]* | ★★★★ | ★ | ★★ | 7 |
| Hirpa et al. [48] | ★★★ | ★★ | ★★★ | 8 |
| Shariff et al. [52] | ★★★ | - | ★★★ | 6 |
| Jitmuang et al. [53] | ★★★★ | ★★ | ★★★ | 9 |
| Chuchottaworn et al. [54] | ★★★★ | ★ | ★★★ | 8 |
| Elmi et al. [55] | ★★ | ★★ | ★★★ | 7 |
| Mulisa et al. [56] | ★★★★ | ★★ | ★ | 7 |
| Mulu et al. [57] | ★★★★ | - | ★★★ | 7 |
| Gunther et al. [58] | ★★★★ | ★★ | ★ | 7 |
| Salindri et al. [62]* | ★★★★ | ★ | ★ | 6 |
| Lee et al. [63] | ★★ | - | ★★★ | 5 |
| Assefa et al. [64] | ★★ | ★★ | ★★★ | 7 |
| Workicho et al. [65] | ★★★ | ★★ | ★★★ | 8 |
| Gobena et al. [68] | ★★★ | - | ★★★ | 6 |
| Dessisa et al. [71] | ★★ | - | ★★★ | 5 |
| Gaborit et al. [72] | ★★ | ★★ | ★★★ | 7 |
| Alene et al. [73] | ★★★ | ★★ | ★★★ | 8 |
| Okethwangu et al. [76] | ★★ | ★ | ★ | 4 |
| Fikre et al. [77] | ★★★ | - | ★★★ | 6 |
| Elduma [78] | ★ ★ ★ | - | ★ | 4 |
| Arroyo et al. [79]* | ★★★★ | - | ★ | 5 |
| Hirama et al. [80]* | ★★★★ | ★★ | ★★ | 8 |

| **Cross-sectional Study** | | | | |
| --- | --- | --- | --- | --- |
| Study | Selection | Comparability | Exposure | Score |
| Brito et al. [28] | ★★★★ | ★★ | ★★★ | 9 |
| Sangare et al. [30] | ★★★ | - | ★★★ | 6 |
| Sangare et al. [31] | ★★★ | - | ★★ | 5 |
| Gudo et al. [32] | ★★★★ | - | ★★ | 6 |
| Vadwai et al. [33] | ★ | - | ★★ | 3 |
| Macedo et al. [34] | ★★★★ | - | ★★★ | 7 |
| Padilla et al. [35] | ★★★★★ | ★★ | ★★★ | 10 |
| van Halsema et al. [36] | ★★★★★ | - | ★★★ | 8 |
| Tesseme et al. [38] | ★★ | - | ★★★ | 5 |
| Coelho et al. [39] | ★★★ | - | ★★ | 5 |
| Ulmasova et al. [40] | ★★★★ | - | ★★★ | 7 |
| Minion et al. [41] | ★★★★ | - | ★★★ | 7 |
| Sethi et al. [42] | ★★★★ | ★★ | ★★★ | 9 |
| van Den Hof et al. [43] | ★★★ | ★★ | ★★★ | 8 |
| Lukoye et al. [44] | ★★★★ | - | ★★★ | 7 |
| Hang et al. [45] | ★★ | ★ | ★★★ | 6 |
| Skrahina et al. [46] | ★★★★★ | ★★ | ★★★ | 10 |
| Mor et al. [49] | ★★★★ | ★ | ★★★ | 8 |
| Post et al. [50] | ★★★ | - | ★★ | 5 |
| Metcalfe et al, [51] | ★★ | ★ | ★★ | 5 |
| Ershova et al. [59] | ★★★ | - | ★★★ | 6 |
| Abdella et al. [60] | ★★★ | ★ | ★★★ | 7 |
| Tadasse [61] | ★★★★ | ★★ | ★ | 7 |
| Sinha et al. [66] | ★★ | - | ★★★ | 5 |
| Mesfin et al. [67] | ★★★ | ★★ | ★★★ | 8 |
| Kusumawati et al. [69] | ★★★★ | - | ★★★ | 7 |
| Pavlenko et al. [70] | ★★★★ | ★★ | ★★★ | 9 |
| Baya et al. [74] | ★★★★ | ★★ | ★★★ | 9 |
| Zurcher et al. [75] | ★★★ | ★ | ★★ | 6 |
| Chen et al. [81] | ★★★★ | - | ★★ | 6 |

**Adapted Newcastle Ottawa Quality Assessment Scale**

**Case-Control Studies [1, 2]**

Note: A study can be awarded a maximum of one point for each numbered item with the Selection and Exposure categories. A maximum of two points can be given for Comparability.

**Selection (maximum 4)**

**1.** Is the case definition adequate? (Multidrug resistant tuberculosis)

a. Requires some independent validation (in which diagnostic method it was ascertained)*

b. Clinical/Hospital/Medical record*

c. Self-reported with no reference to primary record

d. No description

**2.** Representativeness of the cases

a. Consecutive or obviously representative series of cases *

b. Potential for selection biases or not stated

**3.** Selection of controls

a. Community controls from source population*

b. Clinical controls if clinical source of population (same as cases)*

c. Not extracted from same source population

d. No description

**4.** Definition of controls

a. No history of disease at endpoint*

b. No description of source

**Comparability (maximum 2)**

**1.** Comparability of cases and controls on the basis of the design or analysis

a. Study controls for the variable: Previous tuberculosis treatment history*

b. Study controls for any additional socio-demographic factor (e.g. age, sex etc.) *

**Exposure (maximum 3)**

**1.** Ascertainment of exposure (HIV status)

a. Requires some independent validation (in which diagnostic method it was ascertained)*

b. Clinical/Hospital/Medical record*

c. Self-reported with no reference to primary record

d. No description

**2.** Same method of ascertainment for cases and controls

a. Yes *

b. No

**6.** Non-response rate

a. Same rate for both groups *

b. Non-response or missing values should be <20% and accounted for in analysis.

c. Non respondents described

d. rate different and no designation

**Cohort Studies [1, 2]**

Note: A study can be awarded a maximum of one point for each numbered item with the Selection and Exposure categories. A maximum of two points can be given for Comparability.

**Selection (maximum 4)**

**1.** Representativeness of the cohort

a. Truly representative of the average in the target population (random sample or whole)*

b. Somewhat representative of the average in the target population (purposive sampling of representative hospital or healthcare facilities or evidence that the sample is representative of the source population)*

c. Selected group of users/convenient sampling

d. No description of the derivation of the cohort

**2.** Selection of the non-exposed cohort

a. Drawn from the same community as the exposed cohort or hospitals serving the same population as cohort *

b. Drawn from a different source

c. No description of the derivation of the non-exposed cohort

**3.** Ascertainment of exposure (HIV status)

a. Requires some independent validation (in which diagnostic method it was ascertained)*

b. Clinical/Hospital/Medical record*

c. Self-reported with no reference to primary record

d. No description

**4.** Demonstration that outcome of interest (Multidrug resistant tuberculosis) was accounted for or not present at start of study

a. Yes *

b. No

**Comparability (maximum 2)**

**1.** Comparability of cohorts on the basis of the design or analysis

a. Study controls for the variable: Previous tuberculosis treatment history*

b. Study controls for any additional socio-demographic factor (e.g. age, sex etc.) *

**Outcome (maximum 3)**

**1.** Ascertainment of outcome (Multidrug resistant tuberculosis)

a. Requires some independent validation (in which diagnostic method it was ascertained)*

b. Clinical/Hospital/Medical record*

c. Self-reported with no reference to primary record

d. No description

**2.** Was follow-up long enough for outcomes to occur?

a. Yes (>1 year)*

b. No

**3.** Adequacy of follow-up of cohorts

a. Complete follow-up – all subjects accounted for *

b. Subjects lost to follow-up unlikely to introduce bias – small number lost (<20%) or attrition described and accounted for in analysis *

c. Follow up rate not adequate and no description of those lost

d. No statement

**Cross-Sectional Studies [3]**

**Selection (maximum 5)**

**1.** Representativeness of the sample

a. Truly representative of the average in the target population (random sample or whole population) *

b. Somewhat representative of the average in the target population (purposive sampling of representative schools or evidence that the sample is representative of the source population) *

c. Selected group of users/convenience sampling

d. No description of the sampling strategy

**2.** Sample size

a. Justified and satisfactory*

b. Adequately powered to detect a difference (at least 10 events per variable in multivariate analyses)*

c. Not justified

**3.** Non-respondents

a. Comparability between respondents and non-respondents characteristics is established, and the response rate is satisfactory (>80%)*

b. The response rate is unsatisfactory, or the comparability between respondents and non-respondents is unsatisfactory

c. No description of the response rate or the characteristics of the responders & non-responders

**4.** Ascertainment of the exposure (HIV status)

a. Requires some independent validation (in which diagnostic method it was ascertained)**

b. Clinical/Hospital/Medical record*

c. Self-reported with no reference to primary record

d. No description

**Comparability (maximum 2)**

**1.** The subjects in different outcome groups are comparable, based on the study design or analysis. Confounding factors are controlled

a. Study controls for the variable: Previous tuberculosis treatment history*

b. Study controls for any additional socio-demographic factor (e.g. age, sex etc.) *

**Outcome (maximum 3)**

**1.** Assessment of the outcome (Multidrug resistant tuberculosis)

a. Requires some independent validation (in which diagnostic method it was ascertained)**

b. Clinical/Hospital/Medical record*

c. Self-reported with no reference to primary record

d. No description

**2.** Statistical test

a. The statistical test used to analyze the data is clearly described and appropriate, and the measurement of the association is presented as either an OR, CI and P value or a beta coefficient, SE and P value*

b. The statistical test is not appropriate, not described or incomplete

Note: In our scale, we included two stars for the ascertainment of outcome and exposure if the method of determination is described in the study. If any clinical or hospital record is available, then one star is being given. Such studies are observational and performed in hospital mostly.

**Adapted from previous published literature:**

1. Ottawa Hospital Research Institute. http://www.ohri.ca/programs/clinical_epidemiology/oxford.asp (accessed Oct 23, 2019).
2. Epstein S, Roberts E, Sedgwick R, *et al.* Poor school attendance and exclusion: a systematic review protocol on educational risk factors for self-harm and suicidal behaviours. *BMJ Open* 2018; **8**: e023953.
3. Herzog R, Álvarez-Pasquin MJ, Díaz C, Del Barrio JL, Estrada JM, Gil Á. Are healthcare workers’ intentions to vaccinate related to their knowledge, beliefs and attitudes? A systematic review. *BMC Public Health* 2013; **13**: 154.
