# Supplementary material for: HIV infection and multidrug resistant tuberculosis: a systematic review and meta-analysis
Source: BMC Infect Dis. 2021 Jan 11;21:51. doi: 10.1186/s12879-020-05749-2 (PMC7802168; doi:10.1186/s12879-020-05749-2)
Supplement: Supplementary file 4 — Additional file 4. References of studies excluded after full-text review. [file 12879_2020_5749_MOESM4_ESM.docx]

**EXCLUDED ARTICLE REFERENCES:**

1. Gallo JF, Pinhata JMW, Simonsen V, Galesi VMN, Ferrazoli L, Oliveira RS. Prevalence, associated factors, outcomes and transmission of extensively drug-resistant tuberculosis among multidrug-resistant tuberculosis patients in São Paulo, Brazil: a cross-sectional study. *Clin Microbiol Infect* 2018; **24**: 889–95.
2. Abebe G, Abdissa K, Abdissa A, *et al.* Relatively low primary drug resistant tuberculosis in southwestern Ethiopia. *BMC Res Notes* 2012; **5**: 225.
3. Brust JCM, Berman AR, Zalta B, *et al.* Chest radiograph findings and time to culture conversion in patients with multidrug-resistant tuberculosis and HIV in Tugela Ferry, South Africa. *PLoS ONE* 2013; **8**: e73975.
4. Saldanha N, Runwal K, Ghanekar C, Gaikwad S, Sane S, Pujari S. High prevalence of multi drug resistant tuberculosis in people living with HIV in Western India. *BMC Infectious Diseases* 2019; **19**: 391.
5. Isaakidis P, Das M, Kumar AMV, *et al.* Alarming levels of drug-resistant tuberculosis in HIV-infected patients in metropolitan Mumbai, India. *PLoS ONE* 2014; **9**: e110461.
6. Gandhi NR, Shah NS, Andrews JR, *et al.* HIV coinfection in multidrug- and extensively drug-resistant tuberculosis results in high early mortality. *Am J Respir Crit Care Med* 2010; **181**: 80–6.
7. Vella V, Racalbuto V, Guerra R, *et al.* Household contact investigation of multidrug-resistant and extensively drug-resistant tuberculosis in a high HIV prevalence setting. *Int J Tuberc Lung Dis* 2011; **15**: 1170–5, i.
8. Kuksa L, Riekstina V, Leimane V, *et al.* Multi- and extensively drug-resistant tuberculosis in Latvia: trends, characteristics and treatment outcomes. *Public Health Action* 2014; **4**: S47-53.
9. Joh JS, Hong HC, Jeong IA, *et al.* Proportion of multidrug-resistant tuberculosis in human immunodeficiency virus/mycobacterium tuberculosis co-infected patients in Korea. *J Korean Med Sci* 2012; **27**: 1143–6.
10. Yuan X, Zhang T, Kawakami K, *et al.* Genotyping and clinical characteristics of multidrug and extensively drug-resistant tuberculosis in a tertiary care tuberculosis hospital in China. *BMC Infect Dis* 2013; **13**: 315.
11. van Zeller M, Monteiro R, Ramalho J, Almeida I, Duarte R. Multidrug resistant tuberculosis diagnosed by synovial fluid analysis. *Rev Port Pneumol* 2012; **18**: 247–50.
12. Muñoz-Torrico M, Caminero-Luna J, Migliori GB, *et al.* Diabetes is Associated with Severe Adverse Events in Multidrug-Resistant Tuberculosis. *Arch Bronconeumol* 2017; **53**: 245–50.
13. Cai X, Zhang D, Yan Y, Tan D, Xu Y. [Meta-analysis on risk factors of multidrug resistant tuberculosis in China]. *Zhonghua Liu Xing Bing Xue Za Zhi* 2015; **36**: 1424–9.
14. Huai P, Huang X, Cheng J, *et al.* Proportions and Risk Factors of Developing Multidrug Resistance Among Patients with Tuberculosis in China: A Population-Based Case-Control Study. *Microb Drug Resist* 2016; **22**: 717–26.
15. Kurup R, George C. Detection of drug resistant Mycobacterium tuberculosis among patients with and without HIV infection in a rural setting. *West Indian Med J* 2013; **62**: 122–6.
16. Range N, Friis H, Mfaume S, *et al.* Anti-tuberculosis drug resistance pattern among pulmonary tuberculosis patients with or without HIV infection in Mwanza, Tanzania. *Tanzan J Health Res* 2012; **14**: 243–9.
17. Kehinde AO, Adebiyi EO, Salako AO, *et al.* Drug resistance profiles of new- and previously treated patients with pulmonary tuberculosis in Ibadan, Nigeria. *Afr J Med Med Sci* 2016; **45**: 67–73.
18. O’Grady J, Bates M, Chilukutu L, *et al.* Evaluation of the Xpert MTB/RIF assay at a tertiary care referral hospital in a setting where tuberculosis and HIV infection are highly endemic. *Clin Infect Dis* 2012; **55**: 1171–8.
19. Trinh QM, Nguyen HL, Do TN, *et al.* Tuberculosis and HIV co-infection in Vietnam. *Int J Infect Dis* 2016; **46**: 56–60.
20. Muchena G, Shambira G, Masuka N, *et al.* Determinants of multidrug resistance among previously treated tuberculosis patients in Zimbabwe, 2014. *Int J Tuberc Lung Dis* 2017; **21**: 1167–72.
21. Raazi J, Prakash S, Parveen K, Shaikh S. Risk factors of multi-drug resistant tuberculosis in urban Allahabad, India. *International Journal Of Community Medicine And Public Health* 2017; **4**: 2383–8.
22. Lema NA, Majigo M, Mbelele PM, Abade A, Matee MI. Risk factors associated with multidrug resistant tuberculosis among patients referred to Kibong’oto Infectious Disease Hospital in northern Tanzania. *Tanzan J Health Res* 2016; **18**: 1–8.
23. Balaji V, Daley P, Anand AA, *et al.* Risk factors for MDR and XDR-TB in a tertiary referral hospital in India. *PLoS ONE* 2010; **5**: e9527.
24. Falzon D, Mirzayev F, Wares F, *et al.* Multidrug-resistant tuberculosis around the world: what progress has been made? *Eur Respir J* 2015; **45**: 150–6.
25. Resistance to first‐line tuberculosis drugs in three cities of Nigeria
26. Minime-Lingoupou F, Manirakiza A, Yango F, Zandanga G, Le Faou A, Rigouts L. Relatively low primary resistance to anti-tuberculosis drugs in Bangui and Bimbo, Central African Republic. *Int J Tuberc Lung Dis* 2011; **15**: 657–61.
27. Bazira J, Asiimwe BB, Joloba ML, Bwanga F, Matee MI. Use of the GenoType® MTBDRplus assay to assess drug resistance of Mycobacterium tuberculosis isolates from patients in rural Uganda. *BMC Clin Pathol* 2010; **10**: 5.
28. Ali MH, Alrasheedy AA, Hassali MA, Kibuule D, Godman B. Predictors of Multidrug-Resistant Tuberculosis (MDR-TB) in Sudan. *Antibiotics (Basel)* 2019; **8**.
29. Calver AD, Falmer AA, Murray M, *et al.* Emergence of increased resistance and extensively drug-resistant tuberculosis despite treatment adherence, South Africa. *Emerging Infect Dis* 2010; **16**: 264–71.
30. Mekonnen F, Tessema B, Moges F, Gelaw A, Eshetie S, Kumera G. Multidrug resistant tuberculosis: prevalence and risk factors in districts of metema and west armachiho, Northwest Ethiopia. *BMC Infect Dis* 2015; **15**: 461.
31. Farazi A, Sofian M, Zarrinfar N, Katebi F, Hoseini SD, Keshavarz R. Drug resistance pattern and associated risk factors of tuberculosis patients in the central province of Iran. *Caspian J Intern Med* 2013; **4**: 785–9.
32. Demile B, Zenebu A, Shewaye H, Xia S, Guadie A. Risk factors associated with multidrug-resistant tuberculosis (MDR-TB) in a tertiary armed force referral and teaching hospital, Ethiopia. *BMC Infect Dis* 2018; **18**: 249.
33. Fox L, Kramer MR, Haim I, Priess R, Metvachuk A, Shitrit D. Comparison of isoniazid monoresistant tuberculosis with drug-susceptible tuberculosis and multidrug-resistant tuberculosis. *Eur J Clin Microbiol Infect Dis* 2011; **30**: 863–7.
34. Sharma P, Lalwani J, Pandey P, Thakur A. Factors Associated with the Development of Secondary Multidrug-resistant Tuberculosis. *Int J Prev Med* 2019; **10**: 67.
35. Mesfin YM, Hailemariam D, Biadgilign S, Biadglign S, Kibret KT. Association between HIV/AIDS and multi-drug resistance tuberculosis: a systematic review and meta-analysis. *PLoS ONE* 2014; **9**: e82235.
36. Suchindran S, Brouwer ES, Van Rie A. Is HIV infection a risk factor for multi-drug resistant tuberculosis? A systematic review. *PLoS ONE* 2009; **4**: e5561.
37. Berhan A, Berhan Y, Yizengaw D. A meta-analysis of drug resistant tuberculosis in Sub-Saharan Africa: how strongly associated with previous treatment and HIV co-infection? *Ethiop J Health Sci* 2013; **23**: 271–82.
38. Girum T, Muktar E, Lentiro K, Wondiye H, Shewangizaw M. Epidemiology of multidrug-resistant tuberculosis (MDR-TB) in Ethiopia: a systematic review and meta-analysis of the prevalence, determinants and treatment outcome. *Trop Dis Travel Med Vaccines* 2018; **4**: 5.
39. Pradipta IS, Forsman LD, Bruchfeld J, Hak E, Alffenaar J-W. Risk factors of multidrug-resistant tuberculosis: A global systematic review and meta-analysis. *J Infect* 2018; **77**: 469–78.
40. Lukoye D, Ssengooba W, Musisi K, *et al.* Variation and risk factors of drug resistant tuberculosis in sub-Saharan Africa: a systematic review and meta-analysis. *BMC Public Health* 2015; **15**: 291.
41. Zhao Y, Xu S, Wang L, *et al.* National survey of drug-resistant tuberculosis in China. *N Engl J Med* 2012; **366**: 2161–70.
42. Abate D, Taye B, Abseno M, Biadgilign S. Epidemiology of anti-tuberculosis drug resistance patterns and trends in tuberculosis referral hospital in Addis Ababa, Ethiopia. *BMC Res Notes* 2012; **5**: 462.
43. Abouyannis M, Dacombe R, Dambe I, *et al.* Drug resistance of Mycobacterium tuberculosis in Malawi: a cross-sectional survey. *Bull World Health Organ* 2014; **92**: 798–806.
44. Heysell SK, Thomas TA, Gandhi NR, *et al.* Blood cultures for the diagnosis of multidrug-resistant and extensively drug-resistant tuberculosis among HIV-infected patients from rural South Africa: a cross-sectional study. *BMC Infect Dis* 2010; **10**: 344.
45. Gómez-Gómez A, Magaña-Aquino M, López-Meza S, *et al.* Diabetes and Other Risk Factors for Multi-drug Resistant Tuberculosis in a Mexican Population with Pulmonary Tuberculosis: Case Control Study. *Arch Med Res* 2015; **46**: 142–8.
46. Aznar ML, Rando-Segura A, Moreno MM, *et al.* Prevalence and risk factors of multidrug-resistant tuberculosis in Cubal, Angola: a prospective cohort study. *Int J Tuberc Lung Dis* 2019; **23**: 67–72.
47. Banu S, Rahman MT, Ahmed S, *et al.* Multidrug-resistant tuberculosis in Bangladesh: results from a sentinel surveillance system. *Int J Tuberc Lung Dis* 2017; **21**: 12–7.
48. Rifat M, Milton AH, Hall J, *et al.* Development of multidrug resistant tuberculosis in Bangladesh: a case-control study on risk factors. *PLoS ONE* 2014; **9**: e105214.
49. Ahmad AM, Akhtar S, Hasan R, Khan JA, Hussain SF, Rizvi N. Risk factors for multidrug-resistant tuberculosis in urban Pakistan: A multicenter case-control study. *Int J Mycobacteriol* 2012; **1**: 137–42.
50. Stosic M, Vukovic D, Babic D, *et al.* Risk factors for multidrug-resistant tuberculosis among tuberculosis patients in Serbia: a case-control study. *BMC Public Health* 2018; **18**: 1114.
51. Fregona G, Cosme LB, Moreira CMM, *et al.* Risk factors associated with multidrug-resistant tuberculosis in Espírito Santo, Brazil. *Rev Saude Publica* 2017; **51**: 41.
52. He GX, Wang HY, Borgdorff MW, *et al.* Multidrug-resistant tuberculosis, People’s Republic of China, 2007-2009. *Emerging Infect Dis* 2011; **17**: 1831–8.
53. Marahatta SB, Kaewkungwal J, Ramasoota P, Singhasivanon P. Risk factors of multidrug resistant tuberculosis in central Nepal: a pilot study. *Kathmandu Univ Med J (KUMJ)* 2010; **8**: 392–7.
54. Zhang C, Wang Y, Shi G, *et al.* Determinants of multidrug-resistant tuberculosis in Henan province in China: a case control study. *BMC Public Health* 2016; **16**: 42.
55. Jensenius M, Winje BA, Blomberg B, *et al.* Multidrug-resistant tuberculosis in Norway: a nationwide study, 1995-2014. *Int J Tuberc Lung Dis* 2016; **20**: 786–92.
56. Flora MS, Amin MN, Karim MR, *et al.* Risk factors of multi-drug-resistant tuberculosis in Bangladeshi population: a case control study. *Bangladesh Med Res Counc Bull* 2013; **39**: 34–41.
57. Becerra MC, Appleton SC, Franke MF, *et al.* Tuberculosis burden in households of patients with multidrug-resistant and extensively drug-resistant tuberculosis: a retrospective cohort study. *Lancet* 2011; **377**: 147–52.
58. Liang L, Wu Q, Gao L, *et al.* Factors contributing to the high prevalence of multidrug-resistant tuberculosis: a study from China. *Thorax* 2012; **67**: 632–8.
59. Wang K, Chen S, Wang X, *et al.* Factors contributing to the high prevalence of multidrug-resistant tuberculosis among previously treated patients: a case-control study from China. *Microb Drug Resist* 2014; **20**: 294–300.
60. Balabanova Y, Radiulyte B, Davidaviciene E, *et al.* Risk factors for drug-resistant tuberculosis patients in Lithuania, 2002–2008. *European Respiratory Journal* 2012; **39**: 1266–9.
61. Tanrikulu AC, Abakay A, Abakay O. Risk factors for multidrug-resistant tuberculosis in Diyarbakir, Turkey. *Med Sci Monit* 2010; **16**: PH57-62.

# I. Sindani, C. Fitzpatrick, D. Falzon et al., “Multidrug-resistant tuberculosis, somalia, 2010-2011,” Emerging Infectious Diseases, vol. 19, no. 3, pp. 478–480, 2013.

# Hamusse SD, Teshome D, Hussen MS, Demissie M, Lindtjørn B. Primary and secondary anti-tuberculosis drug resistance in Hitossa District of Arsi Zone, Oromia Regional State, Central Ethiopia. *BMC Public Health* 2016; 16: 593.

1. Adane K, Ameni G, Bekele S, Abebe M, Aseffa A. Prevalence and drug resistance profile of Mycobacterium tuberculosis isolated from pulmonary tuberculosis patients attending two public hospitals in East Gojjam zone, northwest Ethiopia. *BMC Public Health* 2015; **15**: 572.
2. Diarra B, Goita D, Tounkara S, *et al.* Tuberculosis drug resistance in Bamako, Mali, from 2006 to 2014. *BMC Infect Dis* 2016; **16**: 714.
3. Chakraborty N, De C, Bhattacharyya S, *et al.* Drug susceptibility profile of Mycobacterium tuberculosis isolated from HIV infected and uninfected pulmonary tuberculosis patients in Eastern India. *Trans R Soc Trop Med Hyg* 2010; **104**: 195–201.
4. Meyssonnier V, Veziris N, Bastian S, Texier-Maugein J, Jarlier V, Robert J. Increase in primary drug resistance of Mycobacterium tuberculosis in younger birth cohorts in France. *Journal of Infection* 2012; **64**: 589–95.
5. Barnett B, Gokhale RH, Krysiak R, *et al.* Prevalence of drug resistant TB among outpatients at an HIV/TB clinic in Lilongwe, Malawi. *Trans R Soc Trop Med Hyg* 2015; **109**: 763–8.
6. Lukoye D, Cobelens FGJ, Ezati N, *et al.* Rates of Anti-Tuberculosis Drug Resistance in Kampala-Uganda Are Low and Not Associated with HIV Infection. *PLOS ONE* 2011; **6**: e16130.
7. Sanchez-Padilla E, Ardizzoni E, Sauvageot D, *et al.* Multidrug- and isoniazid-resistant tuberculosis in three high HIV burden African regions. *Int J Tuberc Lung Dis* 2013; **17**: 1036–42.
8. Cox HS, McDermid C, Azevedo V, *et al.* Epidemic levels of drug resistant tuberculosis (MDR and XDR-TB) in a high HIV prevalence setting in Khayelitsha, South Africa. *PLoS ONE* 2010; **5**: e13901.
9. Yimer SA, Agonafir M, Derese Y, Sani Y, Bjune GA, Holm-Hansen C. Primary drug resistance to anti-tuberculosis drugs in major towns of Amhara region, Ethiopia. *APMIS* 2012; **120**: 503–9.
10. Gomes M, Correia A, Mendonça D, Duarte R. Risk Factors for Drug-Resistant Tuberculosis. *Journal of Tuberculosis Research* 2014; **2**: 111–118.
11. Diandé S, Badoum G, Combary A, *et al.* Multidrug-Resistant Tuberculosis in Burkina Faso from 2006 to 2017: Results of National Surveys. *Eur J Microbiol Immunol (Bp)* 2019; **9**: 23–8.
12. Seyoum B, Demissie M, Worku A, Bekele S, Aseffa A. Prevalence and Drug Resistance Patterns of Mycobacterium tuberculosis among New Smear Positive Pulmonary Tuberculosis Patients in Eastern Ethiopia. *Tuberc Res Treat* 2014; **2014**: 753492.
13. Maru M, Mariam SH, Airgecho T, Gadissa E, Aseffa A. Prevalence of Tuberculosis, Drug Susceptibility Testing, and Genotyping of Mycobacterial Isolates from Pulmonary Tuberculosis Patients in Dessie, Ethiopia. *Tuberc Res Treat* 2015; **2015**: 215015.

# Ombura IP, Onyango N, Odera S, Mutua F, Nyagol J. Prevalence of Drug Resistance Mycobacterium Tuberculosis among Patients Seen in Coast Provincial General Hospital, Mombasa, Kenya. *PLoS One* 2016; 11.

1. Kim H-R, Hwang SS, Kim E-C, *et al.* Risk factors for multidrug-resistant bacterial infection among patients with tuberculosis. *J Hosp Infect* 2011; **77**: 134–7.
2. Dessalegn M, Daniel E, Behailu S, Wagnew M, Nyagero J. Predictors of multidrug resistant tuberculosis among adult patients at Saint Peter Hospital Addis Ababa, Ethiopia. *Pan Afr Med J* 2016; **25**: 5.
3. Gupta A, Nagaraja MR, Kumari P, *et al.* Association of MDR-TB isolates with clinical characteristics of patients from Northern region of India. *Indian J Med Microbiol* 2014; **32**: 270–6.
4. Grandjean L, Gilman RH, Martin L, *et al.* Transmission of Multidrug-Resistant and Drug-Susceptible Tuberculosis within Households: A Prospective Cohort Study. *PLOS Medicine* 2015; **12**: e1001843.
5. Metcalfe JZ, Kim EY, Lin S-YG, *et al.* Determinants of Multidrug-Resistant Tuberculosis Clusters, California, USA, 2004–2007. *Emerg Infect Dis* 2010; **16**: 1403–9.
6. Lohiya A, Suliankatchi Abdulkader R, Rath RS, *et al.* Prevalence and patterns of drug resistant pulmonary tuberculosis in India-A systematic review and meta-analysis. *J Glob Antimicrob Resist* 2020; **22**: 308–16.
7. Singh A, Prasad R, Balasubramanian V, Gupta N. Drug-Resistant Tuberculosis and HIV Infection: Current Perspectives. *HIV AIDS (Auckl)* 2020; **12**: 9–31.
8. Saldanha N, Runwal K, Ghanekar C, Gaikwad S, Sane S, Pujari S. High prevalence of multi drug resistant tuberculosis in people living with HIV in Western India. *BMC Infectious Diseases* 2019; **19**: 391.
9. Ahiarakwem IE, Ekejindu IM, Akujobi CN, Aghanya IN. Multidrug-resistant tuberculosis in Imo State, Southeast, Nigeria. *Nigerian Journal of Clinical Practice* 2020; **23**: 1172.
10. Tembo BP, Malangu NG. Prevalence and factors associated with multidrug/rifampicin resistant tuberculosis among suspected drug resistant tuberculosis patients in Botswana. *BMC Infect Dis* 2019; **19**: 779.
11. Soares VM, Almeida IN de, Figueredo LJ de A, *et al.* Factors associated with tuberculosis and multidrug-resistant tuberculosis in patients treated at a tertiary referral hospital in the state of Minas Gerais, Brazil. *Jornal Brasileiro de Pneumologia* 2020; **46**. DOI:10.36416/1806-3756/e20180386.
12. Tenzin C, Chansatitporn N, Dendup T, *et al.* Factors associated with multidrug-resistant tuberculosis (MDR-TB) in Bhutan: A nationwide case-control study. *PLOS ONE* 2020; **15**: e0236250.
13. Gk A, B H. Trends of Mycobacterium Tuberculosis and rifampicin Resistance in Adigrat General Hospital, Eastern zone of Tigrai, North Ethiopia. 2020; published online May 26. DOI:10.21203/rs.2.21744/v3.
14. Bonin CR, Fochat RC, Leite ICG, *et al.* Analysis of anti-tuberculosis drug resistance and sociodemographic and clinical aspects of patients admitted in a referral hospital. *Einstein (São Paulo)* 2020; **18**. DOI:10.31744/einstein_journal/2020ao4620.
