# Supplementary material for: HIV infection and multidrug resistant tuberculosis: a systematic review and meta-analysis
Source: BMC Infect Dis. 2021 Jan 11;21:51. doi: 10.1186/s12879-020-05749-2 (PMC7802168; doi:10.1186/s12879-020-05749-2)
Supplement: Supplementary file 5 — Additional file 5. Sensitivity analysis. [file 12879_2020_5749_MOESM5_ESM.docx]

Forest plot for studies with more than 1000 participants

Forest plot for studies published in 2016 or later

Forest plot for studies with more than 1000 participants
